# Concentrations and source identification of priority polycyclic aromatic hydrocarbons in sediment cores from south and northeast Thailand

**DOI:** 10.1016/j.heliyon.2022.e10953

**Published:** 2022-10-04

**Authors:** Siwatt Pongpiachan, Danai Tipmanee, Chomsri Choochuay, Woranuch Deelaman, Natthapong Iadtem, Qiyuan Wang, Li Xing, Guohui Li, Yongming Han, Muhammad Zaffar Hashmi, Junji Cao, Apichart Leckngam, Saran Poshyachinda

**Affiliations:** a NIDA Center for Research & Development of Disaster Prevention & Management, School of Social and Environmental Development, National Institute of Development Administration (NIDA), 148 Moo 3, Sereethai Road, Klong-Chan, Bangkapi, Bangkok, 10240, Thailand; b Faculty of Technology and Environment, Prince of Songkla University Phuket Campus 80 M.1 Kathu, Phuket 83120, Thailand; c Faculty of Environmental Management, Prince of Songkla University Hat-Yai Campus, Songkla, 90112, Thailand; d Division of Environmental Science and Technology,Faculty of Science and Technology, Rajamangala University of Technology Phra Nakhon, Bangkok 10800, Thailand; e SKLLQG and Key Lab of Aerosol Chemistry & Physics, Institute of Earth Environment, Chinese Academy of Sciences (IEECAS), Xi'an, 710061, China; f School of Geography and Tourism, Shaanxi Normal University, Xi'an, 710119, China; i Key Lab of Aerosol Chemistry and Physics, SKLLQG, Institute of Earth Environment, Chinese Academy of Sciences, Xi'an, 710061, China; g Department of Chemistry, COMSATS University, Islamabad, Pakistan; h National Astronomical Research Institute of Thailand (Public Organization), 260 Moo 4, T. Donkaew A. Maerim, Chiang Mai, 50180, Thailand

**Keywords:** PAHs, Sediment core, Fresh water in Thailand, Diagnostic binary ratios

## Abstract

In this study, the environmental fate of carcinogenic polycyclic aromatic hydrocarbons (PAHs) in tropical lake sediments and their potential sources have been discussed. 15 PAHs (i.e. ΣPAH) have been investigated in two lakes, namely Songkhla Lake (SKL) and Nong Han Kumphawapi Lake (NHL), which are located at the southern and north-eastern parts of Thailand, respectively. Since these two lakes are registered as important wetlands under the Ramsar convention (United Nations Educational, Scientific and Cultural Organization: UNESCO), the quantitative identification of potential contributors of PAHs is an inevitable analytical tool for launching an evidence-based policy. The ΣPAH concentrations observed in SKL and NHL sediments (*n* = 135) were in the range of 19.4–1,218 ng g^−1^ and 94.5–1,112 ng g^−1^, respectively. While the exponential decline of ΣPAH contents were detected in SKL sediments, NHL showed a trend of enhancing PAH contents with depth. The averaged benzo [a]pyrene (B [a]P) contents of surface sediments in both lakes were much below the value stipulated by the United States Environmental Protection Agency (US-EPA) guidelines for carcinogen risk assessment. Based on numerous multivariate statistical techniques coupled with source apportionment analysis, “biomass burning” and “anthropogenic activities” are two potential contributors of the PAHs detected in the study areas. To achieve the long-term conservation of nature with related ecosystem services and cultural values, it is therefore important to promote decision-making based on ecotoxicological studies of carcinogenic substances.

## 1 Introduction

PAHs are mainly composed of hydrogen and carbon atoms with two or more benzene rings. Over the past few decades, many studies have examined numerous adverse health impacts related to the exposure of PAHs. Some epidemiological studies have described the potential genotoxicity of PAHs on the skin, lung, bladder, liver, and stomach (Armstrong and Gibbs, 2009; Baudouin et al., 2002; Boada et al., 2015; Jiang et al., 2012; Kim et al., 2013; Sarigiannis et al., 2015; Xu et al., 1996). Carcinogenic and mutagenic potencies for various PAHs have also been extensively investigated in several environmental compartments such as aerosols, natural water, soils, and sediments (Apiratikul et al., 2020; ChooChuay et al., 2020a,b,c; Deelaman et al., 2020a,b, 2021; Pongpiachan, 2009; Pongpiachan et al., 2017, 2018a,b).

Recent studies have shown that two source categories (i.e., traffic exhaust and biomass & domestic coal combustion) govern the fluctuation of PAHs in core sediments collected from lakes around the world (Xu et al., 2014). For instance, Unmix, a factor analysis receptor model, indicated that biomass & domestic coal combustion affected a greater proportion of PAHs in the five lakes of Western China (Xu et al., 2014). Both petrogenic and pyrogenic contamination are categorised as two crucial sources of PAHs in the sediments of some lakes, such as Lake Huron in Ontario, Canada and Chini Lake, Malaysia (Bakhtiari et al., 2009; Buell et al., 2021). Three receptor models (i.e., the Principal Component Analysis-Multiple Linear Regression (PCA-MLR) model, Unmix model, and Positive Matrix Factorisation (PMF) model) have also highlighted the importance of traffic exhaust, coal combustion, and wood burning as three main potential sources of PAHs in 29 lake sediments from Taihu Lake, China (Zhang et al., 2012). Diagnostic binary ratios have indicated that oil/coal combustion, vehicle exhaust, and waste burning were three major contributors of PAHs in sediments collected from the Love River and Ho-Jin River, two major urban rivers in Kaohsiung City, Taiwan (Tu et al., 2018). These findings show some agreement with those of another study conducted at the Mai Po Inner Deep Bay Ramsar Site in Hong Kong, which suggested that diesel emission was the most likely contributor of PAHs detected in sediments (Zhao et al., 2012). Although copious investigations have employed different types of source apportionment models to quantitatively identify potential contributors of PAHs in numerous lake sediments around the world, the knowledge of vertical profile of PAH compounds is severely limited, especially in tropical countries, including Thailand. Over the past few years, Thailand has made remarkable progress in social and economic development, moving from a low-income to an upper middle-income country in less than a generation. However, Thailand's rapid economic growth seems to bring more public concerns on various environmental issues particularly the bioaccumulation of carcinogenic substances in pristine places such as wetlands under the Ramsar convention, including SKL and NHL.

A previous study found that the total PAH contents (i.e. summation of 16 US EPA priority PAHs) of core sediment samples, collected in the mangrove swamps of Ma Wan, Hong Kong, increased with depth from 1,300 ng g^−1^ (0–2 cm) to 5,000 ng g^−1^ (10–15 cm) (Li et al., 2009). The exceedingly high percentage contribution of perylene in sediment cores (26–50% (0–12 cm) and 50–77% (12–36 cm)) collected from Chini Lake, Malaysia can be attributed to the activity of termites on trees and shrubs, which generates perylene that is then delivered to the lake by run-off following heavy rain, as well as the *in-situ* formation of perylene in bottom sediments through diagenetic processes (Bakhtiari et al., 2009). A careful investigation of historical trends, spatial distributions, and the potential contributors of PAHs in the sediments of four lakes in Japan has indicated that the impact of anthropogenic activity in the watersheds affects the vertical variation of sediment PAH contents (Guo et al., 2011). The PAH contents of sediments collected from Tokyo Bay increased moderately from the beginning of the early 1900s, reached a peak in the early 1980s, and then declined gradually until the 1990s (Yamashita et al., 2000). Chemical analysis of PAH compositions highlighted the significance of improvements in emission controls in the late 1980s and the early 1990s, which seem to have effectively decreased the inputs of PAHs to the sediments in Tokyo Bay (Yamashita et al., 2000).

Despite extensive studies associated with the chemical characterisation of PAHs in core sediments, knowledge of the spatial and vertical distribution of PAH compounds in lake sediments of Southeast Asia, and of Thailand in particular, is still fragmentary. Thailand, as an upper-middle-income country, has experienced major industrial and social transformations for over half a century. It has fortunately elevated its financial developmental direction from agriculture to export-oriented manufacturing, particularly in the automobile and electronics sector (Asian Development Bank, 2015; Hussey, 1993). Due to this rapid economic growth, it is rational to assume that PAHs may be supplied to the lake by run-off during the southwest monsoon rainy season, which usually occurs from mid-May to October. It is also crucial to emphasise that the increasing number of hotspots in Southeast Asian countries can be considered as another main contributor of PAHs and other toxic pollutants to the environment (Janta et al., 2020; Pongpiachan et al., 2013a; Pongpiachan, 2015; Popovicheva et al., 2017; Reddington et al., 2021). Overall, the main aims of this study are to (*i*) chemically characterise PAHs in sediments collected from the selected locations; (*ii*) use the PAH diagnostic and some multivariate statistical techniques to identify the potential sources of PAH compounds detected in six sediment cores collected from the selected locations.

## 2 Materials & methods

### 2.1 Sampling sites

**Figure 1:**
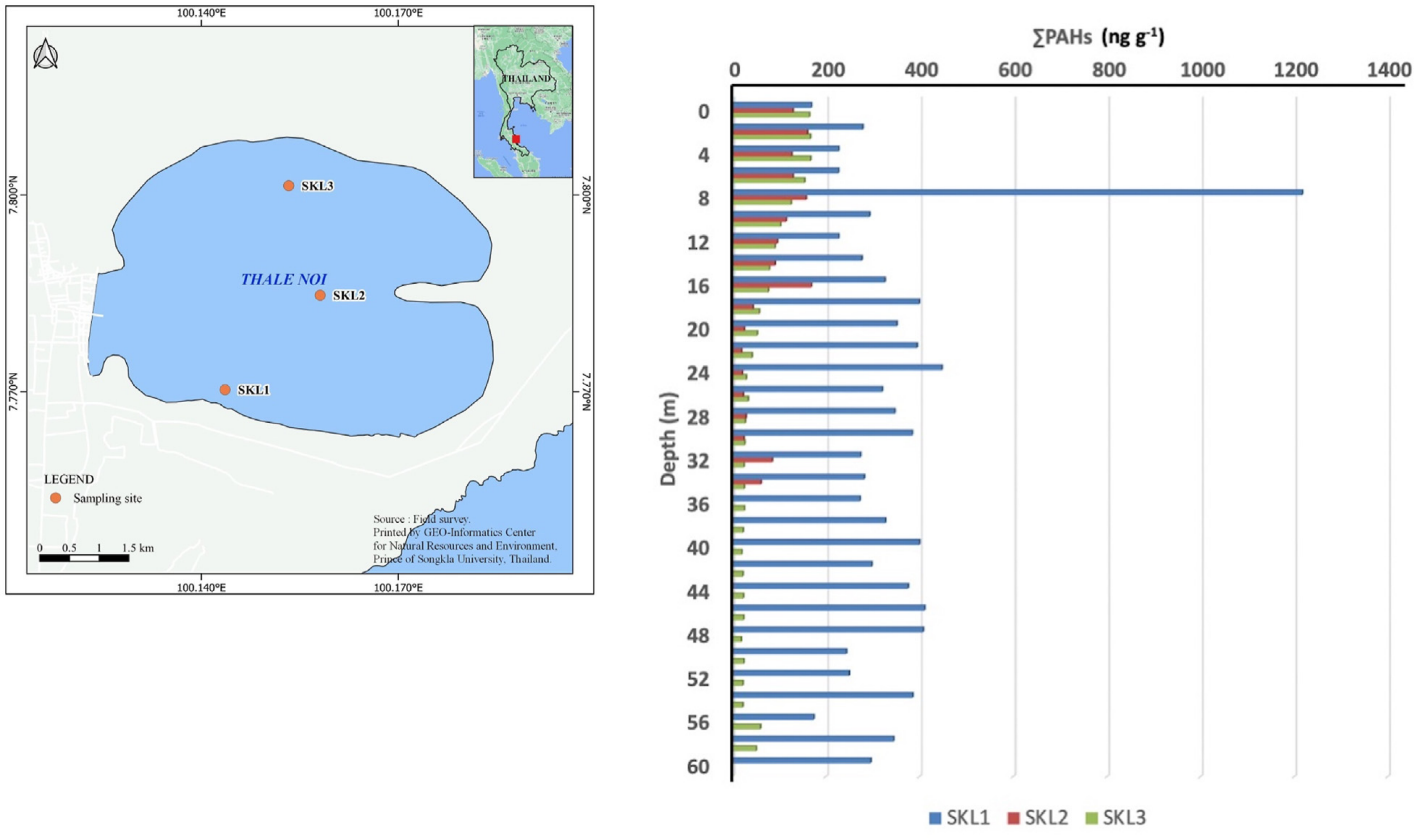
Vertical profile of ∑PAH congeners in lake sediments collected at SKL.

**Figure 2:**
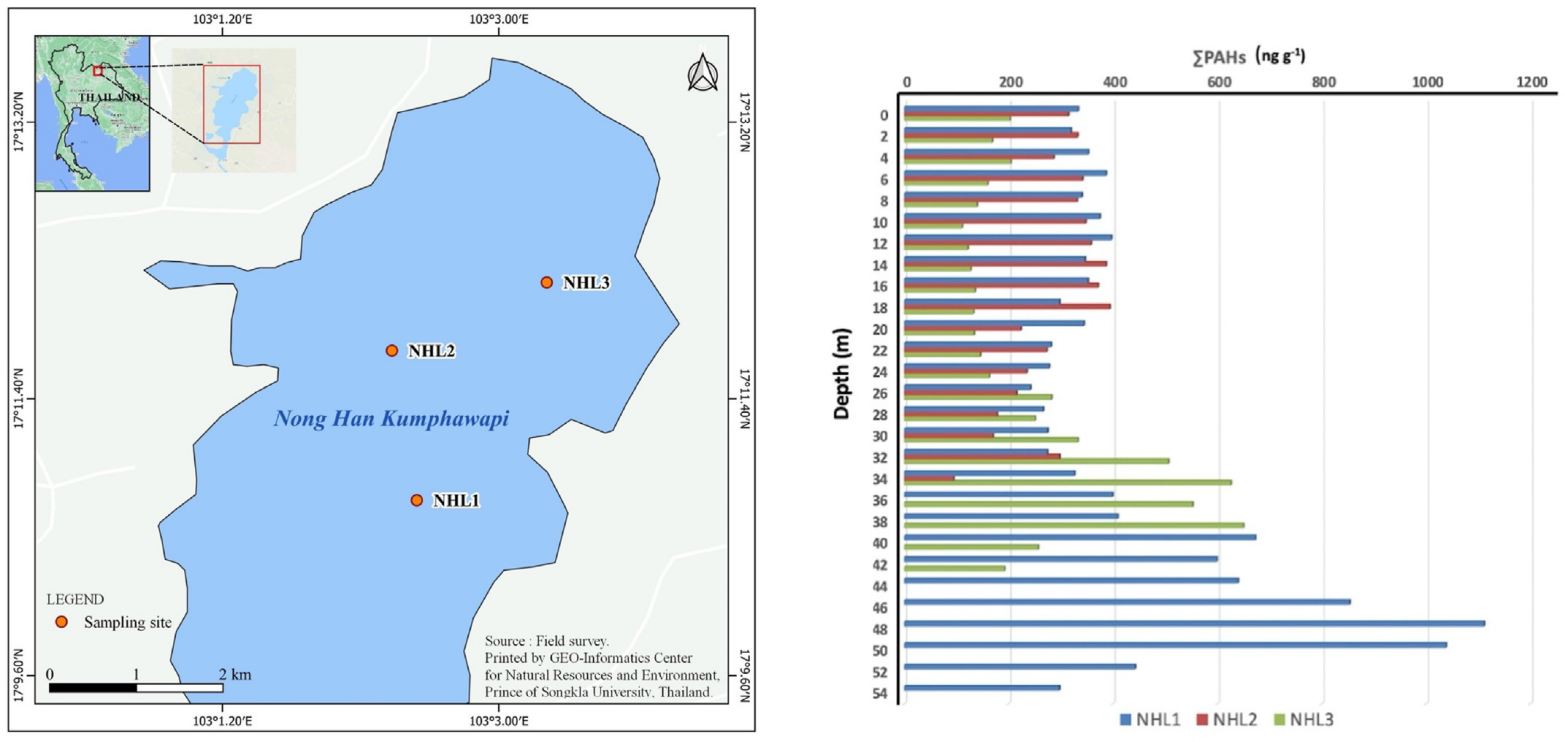
Vertical profile of 15 PAH congeners in lake sediments collected at NHL.

Six sediment cores were collected from SKL and NHL, which are situated in the southern and northeast parts of Thailand, respectively (Figures 1 and 2). The SKL (7°08’′N, 100°07′E; 0–2 m above mean sea level) is the largest coastal shallow lagoon in Thailand, and has a total area of 1,042 km^2^ (Pradit et al., 2010). The SKL is composed of four different parts, namely: Thale Noi (a non-hunting freshwater swamp with a surface area of 28 km^2^), an inner section (with an average depth of 2 m and a surface area of 459 km^2^) that contains brackish water with approximately half the salinity of seawater, a shallow central section (with an average depth of 1 m and a surface area of 377 km^2^), and Thale Sap Songkhla (with an average depth of 1.4 m and a surface area of 182 km^2^) which is connected to the Gulf of Thailand (Pradit et al., 2010). The inner section and shallow central section of the SKL are together known as Thale Luang, and a small group of Irrawaddy dolphins is frequently observed in this water body, adjacent to the Si-Ha Islands of Phatthalung Province (Beasley et al., 2002). A previous study identified medicinal plants in this area (i.e., 95 species belonging to 82 genera in 46 families), many of which (24.3%) have antipyretic properties (Neamsuvan et al., 2015). The SKL is situated in a tropical region, with comparatively high humidity and temperatures throughout the year. The climatic conditions are mostly affected by two monsoons, namely the northeast and southwest monsoons. A previous study reported the average temperature, relative humidity, and average precipitation measured at Songkhla weather station from 1971 to 2000 as 28.1 °C, 76.8%, and 1995 mm, respectively (Ganasut, 2004). The surrounding of the area was contaminated by the activities of human, such as agriculture and residence, open burning for agriculture and forest fires, the most activities tourism and fishing. Additionally, previous source apportionment studies underlined the importance of oil-spill from speed boats as one of the major contributors of PAHs in SKL sediments (Pongpiachan, 2014; Pongpiachan et al., 2013b).

The NHL (17°11′N, 103°02′E; 166 m above mean sea level) is a relatively large natural lake situated in northeast Thailand (Figure 2). A previous study has highlighted the importance of the NHL as a natural freshwater lake containing paleo-climatic historical records spanning more than 10,000 years (Chawchai et al., 2016). The rapid increase in precipitation between c. 7,000 and 6,400 cal. BP enabled the growth and expansion of vegetation in and around the shallow lake (Chawchai et al., 2016). Climate patterns and alterations in the regional hydrological cycle are generally affected by the horizontal transport of water vapor from the Indian and Pacific Oceans and the South China Sea (Loo et al., 2015; Singhrattna et al., 2012). The NHL is annually influenced by atmospheric moisture advection from the Indian and the Pacific Oceans in the middle of the rainy season (i.e., May–October). Additionally, tropical cyclones commonly originate in the South China Sea. The lake morphology and hydro-chemical conditions of the NHL can be described as shallow (<4 m) and circumneutral (pH 6.8), respectively (Chawchai et al., 2016). The areas of open water and wetland were reported as 20 km^2^ and 56 km^2^, respectively (Klubseang, 2011). One of the most notable characteristics of the NHL is its floating plant communities (e.g., *Alocasia macrorhiza*, *Alternanthera philoxeroides*, *Alternanthera sessilis*, *Eichhornia crassipes*, *Ipomea aquatica*, *Ludwigia adscendens*, *Ludwigia octovalis*, *Salvinia cucullata*, and *Saccharum* spp.), which have generated broad herbaceous swamp vegetation (Kealhofer and Penny, 1998; Penny, 1998, 1999). It is worth mentioning that *Nymphaea rubra* (red water lily) is one of the most prosperous species, which generally attracts local and international visitors for sightseeing. Several areas on the surrounding slopes have been cultivated with crops such as sugar cane (*Saccharum officinarum*), castor bean (*Ricinus communis*), peanut (*Arachis hypogaea*), cassava (*Manihot esculenta*), and sesame (*Sesamum indicum*) (Arbhabhirama et al., 1988; Parnwell, 1988). The surrounding of the area was contaminated by the activities of human, such as open burning for agriculture, activities tourism and fishing.

### 2.2 Sample collection

Six sediment cores (i.e. three vertical cores from SKL and another three vertical cores from NHL) were collected at 1^st^ August, 2017 from the SKL. The 58 cm, 32 cm, and 56 cm long sediment cores were obtained from SKL1 (7°46′13″ N, 100°08′37″ E; *n* = 29), SKL2 (7°47′4.9″ N, 100°09′29″ E; *n* = 16), and SKL3 (7°48′05″ N, 100°09′12″ E; *n* = 28), respectively. September 26^th^, 2017 was the date that the 32 cm, 40 cm, and 52 cm long vertical core deposits were gathered from NHL3 (17°12′9.4″ N, 103°03′19″ E; *n* = 16), NHL1 (17°10′44″ N, 103°02′28″ E; *n* = 20), and NHL2 (17°11′43″ N, 103°02′18″ E; *n* = 26), respectively. In this study, the total sub-core samples were 135. It is worth mentioning that the lake water level of all sampling sites varied between 150 and 170 cm (Figures 1 and 2 and Fig. S1–S2). The sediment samples were collected at depths using a transparent polyvinyl chloride plastic tube with a diameter of 10 cm and a length of 150 cm was specially designed for this study, with advantages of simplicity, robustness, and comparative reliability. The core was cut longitudinally and divided into 2 cm *in-situ* immediately after completing the sample collection. The divided preserve samples were stored by freezing at around -20 °C in the aluminium foil. Following sample collection, the core sediments were immediately delivered to the Faculty of Environmental Management, Prince of Songkla University (FEM-PSU), for further PAH analysis.

### 2.3 PAH analysis

A cocktail of 15 PAHs, termed the Norwegian Standard (NS9815: S-4008-100-T) was supplied by Chiron AS (Stiklestadveine 1, N- 7041 Trondheim, Norway). This cocktail comprised phenanthrene (Phe), anthracene (An), fluoranthene (Fluo), pyrene (Pyr), 11H-benzo [a]fluoranthene (11H–B [a]F), 11H-benzo [b]fluoranthene (11H–B [b]F), benzo [a]anthracene (B [a]A), chrysene (Chry), benzo [b]fluoranthene (B [b]F), benzo [k]fluoranthene (B [k]F), benzo [e]pyrene (B [e]P), B [a]P, indeno [1,2,3-cd]pyrene (Ind), dibenz [a,h]anthracene (D [a,h]A), and benzo [g,h,i]perylene (B [g,h,i]P) (each 100 μg mL^−1^ in toluene: unit: 1 × 1 mL). The same company also supplied a mixture of recovery internal standard PAHs (*d*_10_-fluorene; *d*_10_-Fl, *d*_12_-perylene; *d*_12_-Per; each 100 μg mL^−1^ in xylene: unit: 1 × 1 mL). Silica gel (0.040–0.063 mm) and Soxhlet thimbles were supplied by Merck and Whatman (Maidstone, UK), respectively. All materials employed for PAH analysis (e.g., silica gel, glass, and cotton wool, etc.) were placed in a Soxhlet extractor with DCM for 24 h, and left to dry in a desiccator until use. Approximately 10 g of freeze-dried sediment samples were filled in the precleaned thimbles for Soxhlet extraction for 8 h. The volume of DCM solvents was further reduced by pressure distillation to an almost dry state by a combination of both a rotary evaporator and blowing down with a soft nitrogen stream (Gogou et al., 1996). The reduced DCM solutions were subsequently dissolved in 10 ml of *n*-hexane before being applied to the top of a 1.5 g silica gel filled glass column (30 × 0.7 cm diameter) which was previously activated at 150 °C for 3 h. The *n*-hexane solution was consequently fractionised into different polarities of PAHs with the assistance of nitrogen pressure with a flow of 1.4 ml min^−1^ at the bottom of the column (Pongpiachan, 2006). The following solutions were used to elute the numerous compound categories: (*i*) 15 ml *n*-hexane; (*ii*) 15 ml toluene-*n*-hexane (5.6:9.4). After the fractionation process, the eluates were reduced by applying a rotary evaporator followed by evaporation under a soft nitrogen stream. In the current study, 15 individual PAHs were successfully analysed. The PAHs were detected at *m/z* = 178 (Phe and An), *m/z* = 202 (Fluo and Pyr), m/z = 216 (11H–B [a]F and 11H–B [b]F), *m/z* = 228 (B [a]A and Chry), *m/z* = 252 (B [b]F, B [k]F, B [a]P), *m/z* = 254 (B [e]P), *m/z* = 276 (Ind and B [g,h,i]P) and *m/z* = 278 (D [a,h]A), in accordance with Pongpiachan et al., 2017, Pongpiachan et al., 2018a, Pongpiachan et al., 2018b. The initial gas chromatography oven temperature was set at 40 °C with a gradient rate of 10 °*C min*^−1^. All PAH analysis was conducted using the Shimadzu Gas Chromatography Mass Spectrometry (GCMS)-Quadrupole (QP) 2010 Ultra at Bara Scientific Co., Ltd.

### 2.4 Quality assurance/quality control (QA/QC)

#### 2.4.1 Sample collection

All analytical apparatus associated with sediment core sectioning was deliberately precleaned with detergent and water, and subsequently rinsed with methanol (MeOH) and dichloromethane (DCM). Aluminium hard foil knife and spatulas were thoroughly cleaned with distilled water, MeOH, and subsequently DCM. Further QA/QC Control precautions were followed precisely, according to the standard operating procedure for the USGS Reston, Virginia Environmental Organic Geochemistry Laboratory Appendix 3 (https://water.usgs.gov/nrp/biogeochemical-processes-in-groundwater/forms/SOP_LMWOA_05272015_FINAL_Website.pdf). It must be noted that the retrieved sediment columns were preserved in the perpendicular position to avoid any physical interruptions during transport to the FEM-PSU. All sediment samples were freeze-dried to ensure that the target chemicals (i.e., PAHs) were theoretically cooled below the triple point and that sublimation rather than melting would occur in the subsequent steps. The freeze-dried sediment cores were then passed through a 0.15 mm mesh sieve and preserved in a refrigerator at −20 °C in labelled zip lock bags for further chemical analysis. In addition, there was no physical evidence of bioturbation observed in the retrieved sediment columns, suggesting biological limitations in both lakes.

#### 2.4.2 PAH analysis

All details related to QA/QC of the analytical protocol coupled with the optimisation of mass spectrometric conditions were performed in compliance with an earlier report and will not be discussed here (Pongpiachan et al., 2012). The GC temperature programming settings are described in Pongpiachan et al. (2012) and will not be explained here. All accuracies were in the range of 83–115% and relative standard deviation (RSD) were in the range of ±3–8 (see Table S1), in accordance with the extraction of matrix-matched certified National Institute of Standards and Technology (NIST)-Standard Reference Material (SRM) 1941b (*n* = 8). Additionally, the precision of this method, which was calculated as the RSD on duplicate samples, was less than 8%. The concentration of 15 PAHs were quantified by adding the known amount of 3 internal standards into the sediment to obtain the high recovery efficiency from extraction process. Quantity analysis was calculated using relative response factors run in between each batch. The limit of quantitation (LOQ) of the method was the minimum concentration of each 15 PAHs that can be quantified. US Environmental Protection Agency, Test Methods for Evaluating, Solid Waste, SW-846, 3^rd^ edn, Office of Solid Waste (RCRA), Washington DC, 1990. LOQ of each 15 PAHs compound was reported in Table 1. Additionally, the detected concentration in all blanks was below the LOD (see Table 2).

**Table 1:** Limit of Quantification (LOQ) of 15 PAHs in core sediments.

Type of PAHs	LOQ (ng g^−1^)
Phe	0.003
An	0.003
Fluo	0.004
Pyr	0.005
11H–B [a]F	0.020
11H–B [b]F	0.028
B [a]A	0.015
Chry	0.013
B [b]F	0.032
B [k]F	0.032
B [e]P	0.016
B [a]P	0.016
D [a,h]A	0.100
B [g,h,i]P	0.066
Ind	0.058

**Table 2:** Binary ratios of PAH contents collected in lake sediments to its corresponding LOD.

	SKL1 (*n* = 29)	SKL2 (*n* = 16)	SKL3 (*n* = 28)	NHL1 (*n* = 20)	NHL2 (*n* = 26)	NHL3 (*n* = 16)
Phe	7,747	1,457	2,057	8,303	8,297	7,040
An	1,520	223	183	1,690	1,643	1,603
Fluo	7,585	2,010	1,533	7,968	8,215	7,945
Pyr	8,252	1,620	1,206	6,990	6,520	4,912
11H–B [a]F	897	418	184	1,049	738	634
11H–B [b]F	882	174	119	829	724	611
B [a]A	469	135	105	821	479	475
Chry	3,501	338	273	2,394	1,234	1,082
B [b]F	448	201	161	3,175	1,280	1,194
B [k]F	98	51	34	409	169	165
B [e]P	258	193	126	1,801	881	772
B [a]P	4,684	920	503	2,308	1,833	1,308
Ind	507	236	160	1,054	717	612
D [a,h]A	10	5	5	24	15	19
B [g,h,i]P	354	72	47	222	174	154

### 2.5 Statistical analysis

The patterns of PAHs measured in lake sediment cores from SKL and NHL were evaluated using the PPMCC, HCA, and PCA. PPMCC, which is a bivariate correlation, is an indicator of linear correlation between two parameters (e.g. PAHs). In this study, PPMCC was used to determine the correlation coefficients of PAH compounds detected in all lake sediments. HCA, an algorithm commonly used for grouping similar data into clusters, was also used to investigate similarities among the PAHs observed in all lake sediments. PCA is the numerical process of calculating the principal components (PCs) and applying them to conduct a change-of-basis transformation on the data. This method was used to reduce the dimensionality of the data by projecting each data point (i.e. PAHs in lake sediments) onto the main variables, to achieve lower-dimensional data while retaining as much of the data's variation as possible. All statistical analyses were conducted with the software IBM SPSS Statistics, version 25. All the statistical analyses that were referred to above (PPMCC, HCA, and PCA) support the diagnostic ratio that can point to the source of PAHs for each lake.

## 3 Results & discussion

### 3.1 Vertical distribution of PAH concentrations

Table 2 shows the binary ratios of PAHs detected in all lake sediment samples. Since all binary ratios were much greater than one, the reported concentrations can be considered as acceptable. For instance, the binary ratios of Pyr collected at SKL1, SKL2, and SKL3 were 8,252, 1,620, and 1,206, respectively (see Table 2). As illustrated in Table 3, the ΣPAH contents (i.e. the sum of Phe, An, Fluo, Pyr, 11H–B [a]F, 11H–B [b]F, B [a]A, Chry, B [b]F, B [k]F, B [e]P, B [a]P, Ind, D [a,h]A, and B [g,h,i]P) detected in this study were in the range of 19.4–1,218 ng g^−1^ and 94.5–1,112 ng g^−1^ in SKL and NHL sediments, respectively. According to the US-EPA's guidelines for carcinogen risk assessment, B [a]P has been consistently identified as “carcinogenic to humans” by many reliable studies conducted in both animals and humans (EPA, 2005). The average observed B [a]P contents of surface sediments in the NHL and SKL were 26.8 ± 11.5 ng g^−1^ and 27.7 ± 5.69 ng g^−1^, respectively (see Table 4). These values are very close to the guideline value stipulated by the Canadian Council of Ministers of the Environment (31.9 ng g^−1^) and much lower than the values categorised as “effect range low” (430 ng g^−1^), “effect range medium” (1,600 ng g^−1^), and “severe effects level” (1,440,000 ng g^−1^) (see Table 4). The maximum ΣPAHs concentrations in the SKL sediments were detected at depths of 8 cm, 2 cm, and 2 cm with concentrations of 1,218 ng g^−1^, 161 ng g^−1^, and 167 ng g^−1^, respectively (see Figure 1 and Fig. S1). In addition, ΣPAH of SKL and NHL were compared with other area around the world found that were lower than Owen Sound Bay, Canada (2017) 171 times, Lagos lagoon, Nigeria 27 times. While this study is closely the study from Mai Bo Bay, Hongkong (243 ng g^−1^), Conwy Estuary, Unite Kingdom (294 ng g^−1^) and Chapala Lake, Mexico (377 ng g^−1^) (see Table 4).

**Table 3:** Statistical descriptions of PAH congeners (ng g^−1^) in lake sediments collected at Songkhla Lake (SKL) and Nong Han Kumphawapi Lake (NHL).

	SKL1 (*n* = 29)		SKL2 (*n* = 16)		SKL3 (*n* = 28)		NHL1 (*n* = 20)		NHL2 (*n* = 26)		NHL3 (*n* = 16)	
	Average	Std. (%)	Average	Std. (%)	Average	Std. (%)	Average	Std. (%)	Average	Std. (%)	Average	Std. (%)
Phenanthrene	23.2	50.7	4.37	38.9	6.17	32.1	24.9	56.3	24.9	36.6	21.1	24.7
Anthracene	4.56	39.9	.670	65.7	.550	74.5	5.07	34.9	4.93	36.5	4.81	64.2
Fluoranthene	30.3	35.4	8.04	71.8	6.13	95.3	31.9	29.9	32.9	34.8	31.8	51.6
Pyrene	41.3	77.7	8.10	87.8	6.03	95.5	35.0	31.2	32.6	37.9	24.6	54.6
11H-benzo [a]fluoranthene	17.9	54.8	8.35	89.8	3.68	105	21.0	106	14.8	43.7	12.7	114
11H-benzo [b]fluoranthene	24.7	54.0	4.86	85.4	3.33	128	23.2	53.4	20.3	37.3	17.1	105
Benzo [a]anthracene	7.03	48.6	2.02	85.1	1.57	114	12.3	64.7	7.19	29.1	7.13	102
Chrysene	45.5	81.5	4.40	71.6	3.55	90.7	31.1	83.3	16.0	18.0	14.1	102
Benzo [b]fluoranthene	14.3	91.7	6.42	76.3	5.15	87.8	102	101	41.0	24.7	38.2	116
Benzo [k]fluoranthene	3.13	86.6	1.64	70.7	1.08	81.5	13.1	90.6	5.40	18.7	5.28	95.1
Benzo [e]pyrene	4.13	69.7	3.08	74.0	2.01	107	28.8	83.6	14.1	21.8	12.4	95.0
Benzo [a]pyrene	75.0	49.9	14.7	74.7	8.05	106	36.9	37.3	29.3	45.6	20.9	44.2
Indeno [1,2,3-cd]pyrene	29.4	142	13.7	61.6	9.28	78.0	61.1	57.2	41.6	21.1	35.5	72.0
Dibenz [a,h]anthracene	.960	39.6	.490	83.7	.520	110	2.43	43.6	1.54	46.8	1.89	40.7
Benzo [g,h,i]perylene	23.4	333	4.73	44.8	3.09	72.8	14.7	32.0	11.5	33.4	10.2	35.8
∑PAHs	345	85.9	85.6	72.3	60.2	85.3	443	67.1	298	31.7	258	74.7

**Table 4:** Average PAH contents (ng g^−1^) in surface sediments collected at NHL and SKL in comparison with other studies around the world.

Sample Type Region	CCME Guideline	LEL Ontario	SEL Ontario	ERL	ERM	LS NHL	LS SKL	LS Owen Sound Bay	LS Owen Sound Bay	MS Mai Po Bay	LS Baiyangdian Lake	LS Lagos Lagoon	LS 52 Lakes	LS Chapala Lake	LS Michigan Lake	RS Conwy Estuary
Country	Canada	Canada	Canada			Thailand	Thailand	Canada	Canada	Hong Kong	China	Nigeria	China	Mexico	USA	UK
Sampling Year	2008							2015	2017	2004	2009			2012	2011	2017
Ref	[1]	[2]	[2]	[3]	[4]	This study	This study	[2]	[2]	[5]	[6]	[7]	[8]	[9]	[10]	[11]
Phenanthrene	41.9	560	950,000	240	1,500	38.8	8.59	376	4,876	24.6	140	240	5.888–536.115	85.5	70.4	32
Anthracene	46.9	220	370,000	85	1,100	6.20	1.52	69	262	20	137	550	0.287–134.702	17.1	9.1	5
Fluoranthene	111	750	1,020,000	600	5,100	35.6	14.8	580	7,500	41.3	55.6	390	1.319–773.955	49.8	134.1	NV
Pyrene	53	490	850,000	660	2,500	32.9	16.4	472	6,702	50.4	88.9	980	0.765–655.641	93.9	110.3	24
Benzo [a]anthracene	31.7	320	1,480,000	260	1,600	6.54	3.51	247	4,284	25.8	3.52	560	0.601–580.910	1.2	55.8	NV
Chrysene	57.1	340	460,000	380	2,800	13.3	6.49	279	4,107	6.7	3.41	430	0.399–601.450	4.3	75.9	20
Benzo [b]fluoranthene	NV	NV	NV	320	1,880	28.1	13.5	185	4,764	16.3	1.18	4	NV	4.6	28.5	NV
Benzo [k]fluoranthene	NV	240	1,340,000	280	1,620	4.76	2.37	138	2,216	27.5	2.73	0.8	NV	NV	22.5	NV
Benzo [e]pyrene	NV	NV	NV	NV		11.2	6.10	NV	NV	NV	NV	NV	NV	2.6	NV	NV
Benzo [a]pyrene	31.9	370	1,440,000	430	1,600	26.8	27.7	12	4,068	8.3	1.28	510	0.060–791.013	7.9	2.7	30
Indeno [1,2,3-cd]pyrene	NV	200	320,000	NV	NV	40.7	27.3	167	2,709	2.1	NV	2,850	NV	ND	0.61	103
Dibenz [a,h]anthracene	6	60	130,000	430	1,600	1.54	1.37	22	545	2.1	NV	NV	0.127–292.695	ND	6.7	NV
Benzo [g,h,i]perylene	NV	170	320,000	63	260	15.0	11.5	66	2,649	17.9	NV	480	NV	110	0.52	80
Σ_13_PAHs						261.4	141.2	2,613	44,682	243.0	434	6,995		377	517	294
ΣPAHs(3,4)						133.3	51.3	2,023	27,731	168.8	428	3,150		252	456	81
ΣPAHs(5,6)						128.1	89.9	590	16,951	74.2	5.19	3,845		125	62	213

LEL, Lowest Effects Level.

SEL, Severe Effects Level.

ERL, Effect Range Low.

ERM, Effect Range Median.

NV, No value was provided.

ΣPAHs(3,4) Total sum of 3,4 ring PAHs ΣPAHs(5,6) Total sum of 5,6 ring PAHs.

LS, Lake Sediment.

MS, Mudflat Sediment.

RS, River Sediment.

Σ_13_PAHs, Total sum of 13 PAH congeners.

[1] CCME, 2008.

[2] Buell et al. (2021).

[3] Long (1992).

[4] Burton (2002).

[5] Zhao et al. (2012).

[6] Guo et al. (2011).

[7] Benson et al. (2020).

[8] Li et al. (2017).

[9] Ontiveros-Cuadras et al. (2019).

[10] Huang et al. (2014).

[11] Vane et al. (2020).

On the contrary, the minimum concentrations were measured at depths of 0 cm, 22 cm, and 48 cm with concentrations of 169 ng g^−1^, 20.0 ng g^−1^, and 19.4 ng g^−1^, respectively. This indicates that more PAHs had accumulated in subsurface SKL sediments. This phenomenon can be attributed to numerous factors. Several studies highlight leaking gasoline from speed boats as one of the main contributors of PAHs in coastal soils and sediments (Iwegbue et al., 2021; Pongpiachan et al., 2018a). Over the past few years, the SKL has become an ecotourism hotspot, facilitating numerous activities that require water transportation, such as wild fruit collection, bird watching, camping, and water sports (Chaiyakot and Visuthismajarn, 2010). Rapid industrialisation and urbanisation of the Songkhla and its neighbouring provinces has raised public health concerns over the increasing amount of PAHs unintentionally released from rubber-wood burning in rubber sheet production, coupled with increasing traffic emissions, particularly from the diesel exhausts of buses and heavy-duty vehicles (Chaichana et al., 2011; Choosong et al., 2010; Tekasakul et al., 2008; Thumanu et al., 2009).

In NHL sediments, the highest ΣPAH contents were observed at depths of 48 cm, 18 cm, and 38 cm with concentrations of 1,112 ng g^−1^, 394 ng g^−1^, and 650 ng g^−1^, respectively (Figure 2 and Fig. S2). In contrast to the SKL samples, NHL samples exhibited a trend of increasing PAH concentrations with depth. Although the prehistoric record of the area surrounding the NHL is far from complete, a previous study suggested that human occupation of the Kumphawapi district probably intensified at approximately 6,000 years B.P (Penny et al., 1996). White (1995) has indicated that around 2,000 years ago, rice cultivation likely advanced from wild rice gathering to a permanent bunded field system resembling the natural ecological conditions suitable for rice. Although either the burning of agricultural waste (e.g. rice straw) or natural forest fires could be responsible for the increase in PAHs in paleo-sediments collected from the NHL, various depletion mechanisms can play a major role in reducing PAH concentrations in surface sediment layers. Firstly, numerous studies have indicated that endophytic microbes can reduce the concentration of absorbed PAHs when they colonise and grow inside of a plant (Durán et al., 2018; Fu et al., 2020; Ghosh et al., 2018). Microbial degradation and co-metabolism are two main factors which degrade PAHs in the rhizosphere (Shimp et al., 1993). Furthermore, a considerable number of grass species (e.g., *Agropyron smithii, Bouteloua gracilis, Cyanodon dactylon, Elymus Canadensis, Festuca arundinacea*, *Festuca rubra, Melilotus officinalis*, etc.) have been recognised as PAH-degrading vegetation (Christensen-Kirsh, 1996; McCutcheon and Schnoor, 2004). Secondly, the presence of both humic and fulvic acids can greatly affect the leaching capability of PAHs in natural water and thus reduce their concentrations in lake sediments. The apparent water solubility of Pyr was quantitatively evaluated by considering the partitioning of Pyr from water into humic substances (HSs) (Tanaka et al., 1997). The degree of aromaticity of HSs is a controlling factor for enhancing the apparent solubility of Pyr (Tanaka et al., 1997). Over the past few decades, several studies have analysed the impacts of HSs on the apparent water solubility of other PAHs (Laor and Rebhun, 2002; Pongpiachan, 2009; Tremblay et al., 2005). Based on X-ray fluorescence analysis, NHL sediments can be categorised as highly organic- and peat-rich sediments (Chawchai et al., 2016). Hence it is reasonable to interpret the relatively low PAH contents of the NHL surface sediments as an indicator of the coexistence of PAHs and HSs in a peat swamp area. Irrespective of the fact that all detected B [a]P contents were close to the international guideline, the vertical distribution of ΣPAHs concentrations measured at NHL were exponentially increased with depth. On the contrary, the highest ΣPAHs contents were detected at subsurface SKL sediments.

### 3.2 Percentage contribution of PAH contents

**Figure 3:**
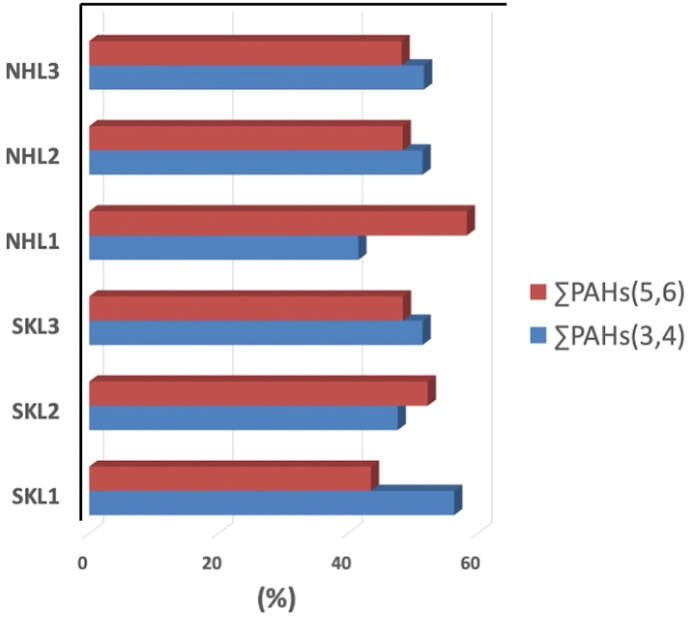
Percentage contribution of the former by low molecular weight PAHs (ΣPAHs (3,4)) and high molecular weight.

As illustrated in Figure 3, the average percentage contributions of ΣPAHs (3,4) detected at SKL and NHL were 52 ± 4.0 % and 49 ± 5.8 %, respectively. These values both are close to 50%, representing a unique feature of this area. As shown in Table 4, the ΣPAHs (3,4) contents detected in sediments from Owen Sound Bay, Canada (77% and 62%), Mai Po Bay, Hong Kong (69%), Baiyangdian Lake, China (99%), Chapala Lake, Mexico (67%), and Michigan Lake, USA (88%) were all greater than 50%. Only a few sediment samples collected at Lagos Lagoon, Nigeria (45%) and Conwy Estuary, UK (28%) have exhibited ΣPAHs (3,4) percentage contributions below 50%. A reassessment of reported spatial and temporal distribution patterns of PAHs in surface terrestrial soils from 27 locations all over the world indicates that there are two distinct PAH patterns, namely a background and an anthropogenic pattern (Wilcke, 2007). The former is governed by low molecular weight PAHs (LMW PAHs), the latter by high molecular weight PAHs (HMW PAHs) (Wilcke, 2007). A more recent review of PAH patterns in global sediments has revealed that the percentage contributions of HMW PAHs normally varies from 56.3 to 93.6% with potential sources related to run-off, atmospheric deposition, and imperfect combustion of fossil fuels (Adeniji et al., 2018). Since the percentage contributions of ΣPAHs(3,4) and ΣPAHs(5,6) detected in both lakes were close to 50%, the sources of PAHs in SKL and NHL can be identified as a mixture of “background” and “human-activities”.

It is also interesting to note that numerous factors can dramatically alter the percentage contributions of ΣPAHs(3,4) and ΣPAHs(5,6). The influence of PAH molecular weights on the soil-plant transfer of PAHs was investigated using three short-life herbaceous species, namely *Eleusine indica*, *Cynodon dactylon*, and *Alternanthera sessilis* (Matsodoum Nguemté et al., 2020). Model and experimental results showed that PAH concentrations can vary significantly between different compartments of plants. For instance, Fluo and Pyr (i.e. LMW PAHs) tend to bioaccumulate in stem and leave compartments while HMW PAHs tend to occur in plant lipids and comparatively higher organic matter areas (Balasubramaniyam, 2015; Matsodoum Nguemté et al., 2020). Because both lakes are surrounded by highly biodiverse herbaceous tropical plants, the impact of plant *ab*sorption on the alteration of percentage contributions of PAHs is undeniable. Several studies have suggested that the soot content of sediments greatly affects the sediment-water partitioning of PAHs in lake and riverine waters (Qiao et al., 2008). In sediment samples taken from six sites in Meiliang Bay, Taihu Lake, China, PAHs showed a high tendency to bind strongly to soot carbon (Qiao et al., 2008). A similar study was conducted in Yimma River Basin, China to investigate the PAH sediment-water partitioning behaviour (Sun et al., 2017). Careful analysis of the octanol/water partitioning coefficient (*K*_ow_) coupled with the organic carbon normalised partitioning coefficient (*K*_oc_) showed that PAH partitioning behaviour was comparatively diverse in numerous locations, which could be attributed to the varying organic carbon contents of different areas (Qin et al., 2014; Sun et al., 2017). A previous study also highlighted the impact of chemical compositions, particularly organic contents, on PAH sorption by suspended particulate matter (SPM). This factor had a far greater impact than other environmental factors such as water temperature, salinity, and dissolved organic matter concentrations (Tremblay et al., 2005). Since the NHL is situated in the middle of a tropical peat swamp forest, we can assume that the majority of PAHs in surface sediments were *ab*sorbed by SPM due to its organic-rich properties. As a consequence, a larger quantity of PAHs might have been transferring from surface sediments to SPM via the sediment-particle portioning process, leading to a subsequent reduction in the amount of PAH contaminants in the surface layers. While the majority of world lake sediments are mainly occupied by ΣPAHs(3,4) with the percentage contribution larger than 50%, ΣPAHs(3,4) and ΣPAHs(5,6) measured in both lakes were close to 50%, indicating that the potential contributors of PAHs in SKL and NHL can be explained as a mixture of “background” and “anthropogenic activities”.

### 3.3 Diagnostic binary ratios of PAHs

Over the past few decades, diagnostic binary ratios have been used for source identification of PAHs in various environmental compartments (ChooChuay et al., 2020a,b,c; Deelaman et al., 2020a,b, 2021; Pongpiachan et al., 2017, 2018a,b; Yunker et al., 2002, 2003, 2011). In this study, An/(Phe + An), Fluo/(Fluo + Pyr), B [a]A/(B [a]A + Chry), B [b + j + k]F/(B [b + j + k]F + B [e]P), and Ind/(Ind + B [g,h,i]P) were used to determine the potential sources of PAH compounds present in sediments from the two lakes. A Ind/(Ind + B [g,h,i]P) ratio of less than 0.2 implies a petroleum source, while a value of 0.2–0.5 indicates a mixture of sources comprising liquid fossil fuel combustion, and any values higher than 0.5 indicate the burning of biomass and coal. It is also interesting to note that other diagnostic binary ratios can also be employed as geochemical proxies to identify biogenic combustion. For instance, if An/(An + Phe), Fluo/(Fluo + Pyr), and B [a]A/(B [a]A + Chry) are greater than 0.1, 0.5, and 0.35, respectively, it suggests a comparatively strong influence from the combustion of grass/wood/coal. Furthermore, B [b + j + k]F/(B [b + j + k]F + B [e]P) values of less than 0.5 indicate a relatively high impact of oil spills.

**Figure 4:**
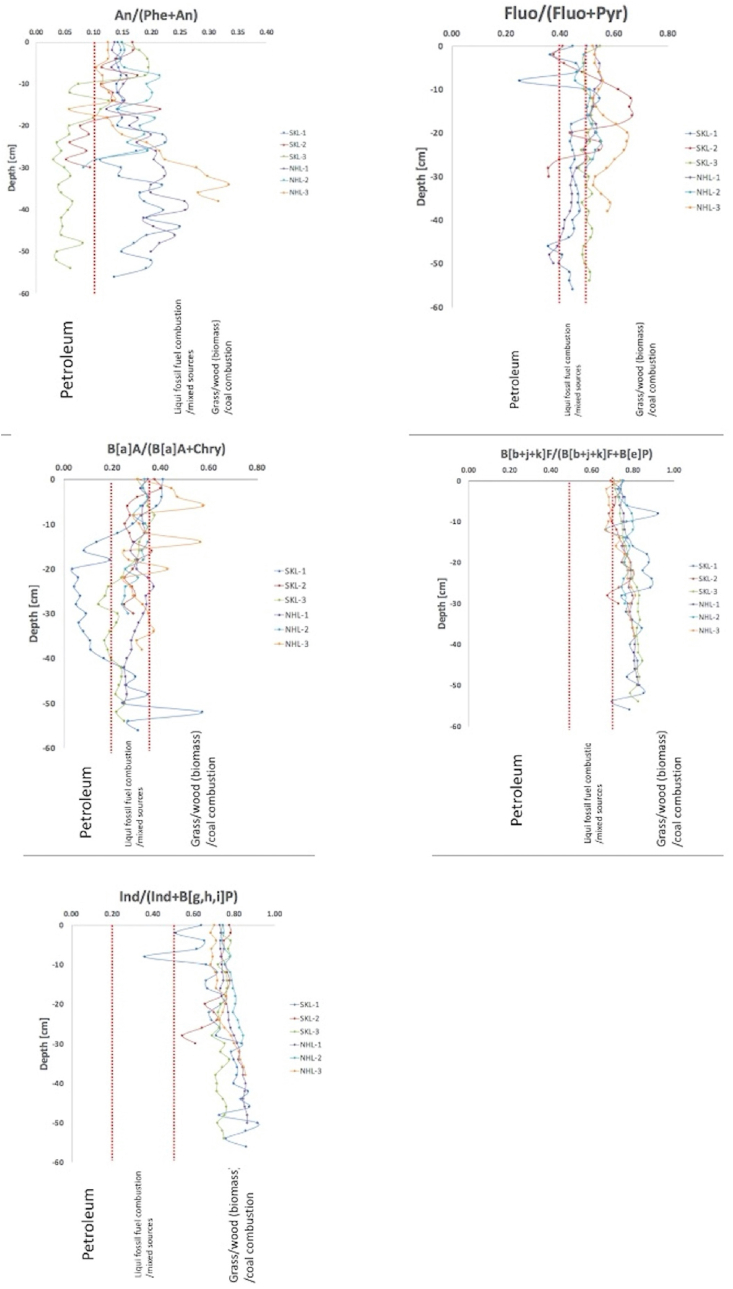
Diagnostic binary ratios of An/(Phe + An), Fluo/(Fluo + Pyr), B [a]A/(B [a]A + Chry), B [b + j + k]F/(B [b + j + k]F + B [e]P), and Ind/(Ind + B [g,h,i]P) in lake sediments collected at SKL and NHL.

As illustrated in Table S2–S5, the computation of four different diagnostic binary ratios of PAHs (i.e. An/(An + Phe), Fluo/(Fluo + Pyr), B [a]A/(B [a]A + Chry), and B [b + j + k]F/(B [b + j + k]F + B [e]P)) were clearly presented. In Figure 4, the samples SKL1 and SKL3 plot in the zone of petroleum, as indicated by An/(Phe + An) and B [a]A/(B [a]A + Chry). It should be noted that no NHL samples were found to plot within the zone of petroleum, as illustrated in vertical plots of all categories of diagnostic binary ratios (see Figure 4). This can be explained by the unique geophysical features of NHL, which is a pristine natural lake that is less impacted by human activities. On the contrary, SKL is greatly affected by both the urbanisation and industrialisation that has it occurred in Songkhla Province over the past few years. Songkhla Province plays a crucial role in the economic and social development of the Southern Thailand, as it is a multicultural financial centre for para-rubber industries and other agronomic crops. Recently, numerous ecotourism activities (e.g. trekking, hiking, kayaking, bird watching etc.) have attracted more domestic and international tourists to the SKL, leading to an increase in water transportation, including passenger speed boats and fishing boats. Accidents when refuelling ships or boats, including small oil slicks from motor vehicle spills, are one of the main contributors of PAHs in SKL sediments.

Despite the obvious evidence of oil-spill-derived PAHs in SKL sediments, the diagnostic binary ratios exhibited some discrepancies, which require further discussion. For instance, the diagnostic binary ratios of (B [b + j + k]F/(B [b + j + k]F + B [e]P) and Ind/(Ind + B [g,h,i]P) indicated that almost all sediment layers contained PAHs originating from biomass and coal combustion. On the contrary, An/(Phe + An) and B [a]A/(B [a]A + Chry) suggested that oil spills are a potential source of PAHs in some SKL sediment samples. Such inconsistencies arise from various factors. Firstly, there is limited data associated with diagnostic binary ratios of PAHs in tropical vegetation, and thus the ratios may not reflect relevant aspects of the real-world situation. In other words, the archive of diagnostic binary ratios for PAH compounds should be revised and re-considered based on aerosol chamber experiments with updated tropical grasses and biomasses. Secondly, several tropical meteorological conditions (e.g. temperature, ultra violet (UV)-light, solar radiation, etc.) can dramatically reduce the atmospheric lifetimes of each PAH and thus alter the diagnostic binary ratios of specific sources. Despite numerous studies relating to both homogeneous and heterogeneous chemical reactions of PAHs with certain trace gaseous species (e.g. the hydroxyl (OH) radical, nitrogen dioxide (NO_2_), nitrate (NO_3_), dinitrogen pentaoxide (N_2_O_5_), a recent study has underlined the importance of acknowledging ambient temperature and relative humidity influences on the atmospheric lifetime of B [a]P (Arey et al., 1989; Atkinson and Arey, 1994; Feilberg et al., 1999; Mu et al., 2018). Thirdly, the application of diagnostic binary ratios assumes that the water-colloid partitioning mechanism of PAHs is negligible. This assumption may be problematic since the aromaticity of HSs in natural water can dramatically affect the sorption mechanism and thus alter the diagnostic binary ratios of PAHs (Tremblay et al., 2005). Although there are many substantial possibilities influencing the effectiveness and reliability of this technique, the geographical remoteness of the NHL can substantially limit the contamination of PAHs, particularly from oil spills. An/(Phe + An) and B [a]A/(B [a]A + Chry) have also indicated the possibility of ecotoxicological damage from oil spills resulting from the promotion of ecotourism activities, especially at Thale Noi Non-Hunting Area, which is a part of the greater Songkhla Lake basin. Overall, the four different diagnostic binary ratios highlighted the overwhelming influence of liquid fossil fuel combustions, biomass (i.e. grass and wood) burning, and coal combustion over the variation of PAH contents in NHL and SKL. In addition, the impact of oil spill can be considered as a minor importance of potential PAH sources, especially in NHL.

### 3.4 PPMCC

The PPMCC has been used for assessing the impacts of potential sources of PAHs in several environmental compartments (ChooChuay et al., 2020a,b,c; Deelaman et al., 2020a,b; Pongpiachan et al., 2017, 2018a,b). In Tables S6-S7, presents the PPMCCs of PAHs in lake sediment samples collected at SKL and NHL. with any values higher than 0.5 highlighted in bold. Certain distinguishing characteristics of the PPMCC can be extracted from Table S6–S7. Firstly, in SKL sediment samples, Phe and An exhibited moderately strong positive correlations with other LMW PAHs detected in the samples. These correlations were quite prominent when compared to those of the NHL samples. Previous studies have shown that Phe and An are mainly found in diesel emissions (de Abrantes et al., 2004; Doretti et al., 1986; Tavares et al., 2004). These findings are in good agreement with those described in the section *Diagnostic binary ratios of PAHs*, indicating that oil spills from diesel boat engines play a crucial role in PAH contamination of SKL sediments. Secondly, Ind, D [a,h]A, and B [g,h,i]P exhibited considerably strong positive correlations with other PAHs in the NHL sediment samples. An earlier investigation demonstrated that these three HMW PAHs were predominantly present in particles emitted from wood combustion (Cereceda-Balic et al., 2012). The maximum concentration of D [a,h]A was also found in a rural household in India, as a consequence of unprocessed biomass combustion for cooking food during a cold period (Bhargava et al., 2004). Thirdly, B [a]A in the NHL samples exhibited strong positive correlations with numerous other PAHs (e.g. B [a]A *vs.* Chry (*R* = 0.97), B [a]A *vs.* B [b]F (*R* = 0.93), B [a]A *vs.* B [b]F (*R* = 0.93), B [a]A *vs.* B [k]F (*R* = 0.94), B [a]A *vs.* B [e]P (*R* = 0.95), B [a]P *vs.* Ind (*R* = 0.97), B [a]A *vs.* D [a,h]A (*R* = 0.72), and B [a]A *vs.* B [g,h,i]P (*R* = 0.70)). An inventory of fine particulate organic compound emissions from residential wood combustion in Portugal underlined the exceedingly high concentrations of B [a]A, which varied from 63 ng g^−1^ to 1,303 ng g^−1^ in eight different wood species, namely maritime pine, golden wattle, holm oak, eucalyptus, olive, cork oak, Portuguese oak, and Briquettes/Pellets (Gonçalves et al., 2012). Overall, we can conclude that biomass combustion is a major governing factor of PAH concentrations in the sediments of a pristine natural lake such as the NHL.

### 3.5 Hierarchical cluster analysis (HCA)

**Figure 5:**
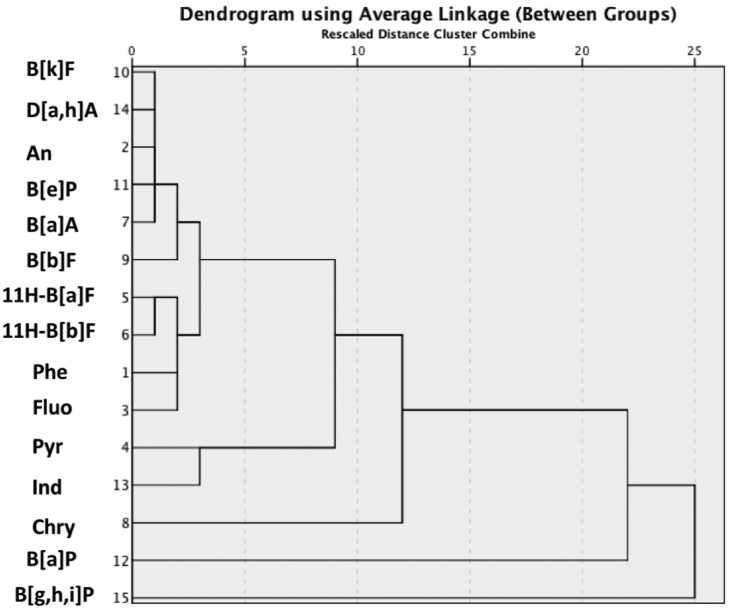
Hierarchical cluster analysis of 15 PAH congeners in lake sediments collected at SKL.

**Figure 6:**
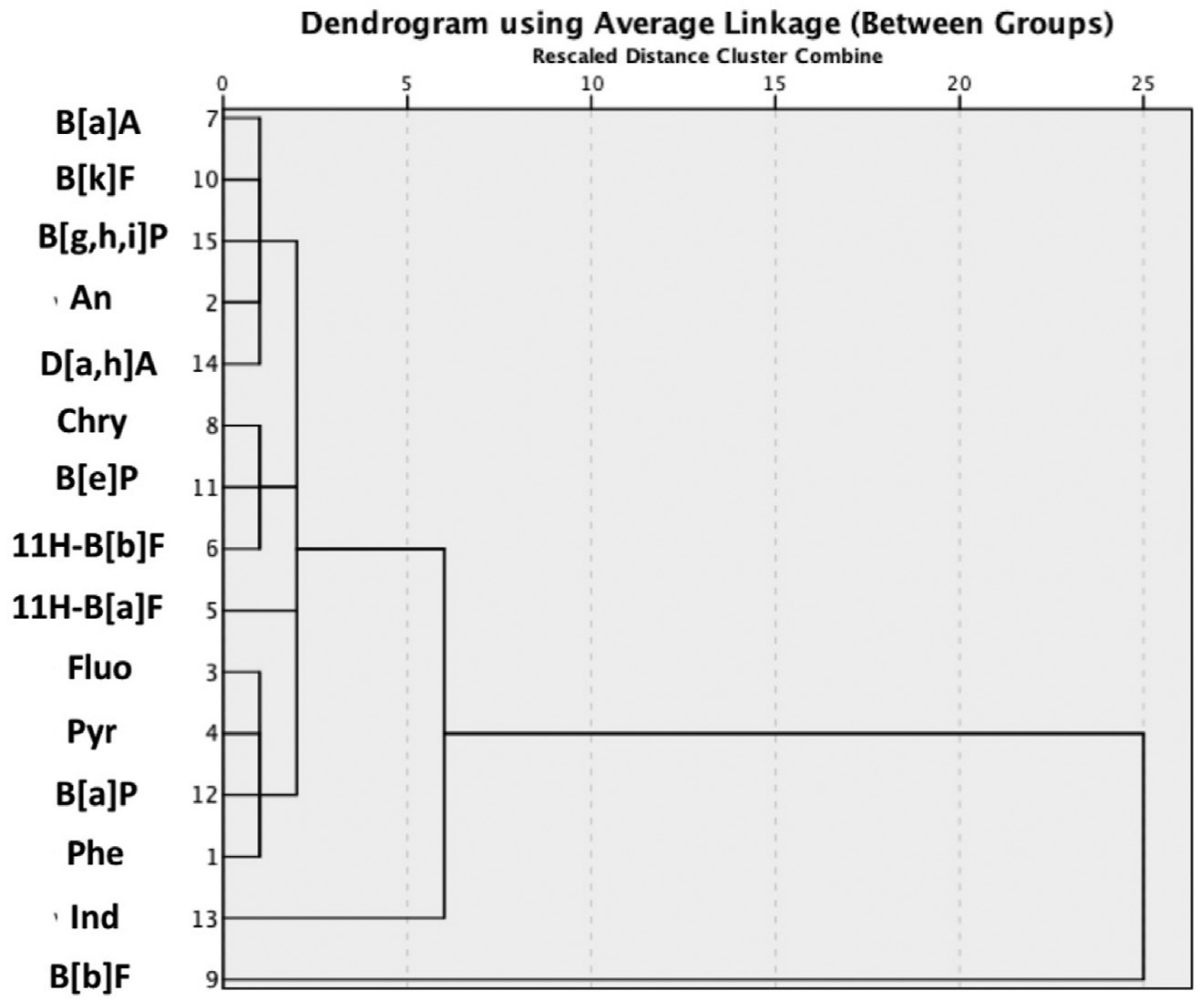
Hierarchical cluster analysis of 15 PAH congeners in lake sediments collected at NHL.

HCA is an advanced multivariate statistical method used in data mining, which is used to construct a hierarchy of clusters. HCA can be categorised into two types namely *agglomerative* (i.e., a bottom-up approach) and *decisive* (i.e., a top-down approach). The former operates by allowing each datapoint to begin as its own cluster and pairs of clusters are then merged as one ascends up the hierarchy, while the latter follows an algorithm of recursive splitting of datapoints from one cluster as one descends down the hierarchy. In this study, the average linkage criteria were employed for selecting which clusters should be merged as a part of the agglomerative process. Dendrograms were constructed by average linkages between groups of PAH contents of sediments collected from the SKL and NHL. These are illustrated in Figures 5 and 6, respectively. It is quite obvious that the dendrogram for the SKL samples has more subclusters than the NHL dendrogram. This indicates a higher degree of dissimilarity of the associated cluster membership for each datapoint detected in SKL sediment samples. For instance, Pyr, Ind, Chry, B [a]P, and B [g,h,i]P were highly deviated from the subcluster of B [k]F, D [a,h]A, An, B [e]P, and B [a]A. A previous chemical characterisation of particulate PAHs in the ambient air of PSU, Hat-Yai city, which is situated approximately 125 km SE of the SKL, showed the comparatively high abundances of Phe, Pyr, B [a]P, Ind, and B [g,h,i]P in total suspended particles (Tekasakul et al., 2008). As previously mentioned in section 3.4, D [a,h]A and B [a]A are mainly found in aerosols emitted from the combustion of biomass and wood (Bhargava et al., 2004; Gonçalves et al., 2012). Hence, it is reasonable to ascribe the moderately deviated subclusters present in the SKL dendrogram to a mixture of highly diversified potential sources of PAHs in Songkhla Province. On the contrary, the NHL dendrogram exhibited a lower degree of deviations, as the majority of PAHs occurred in the same main cluster (Figure 6). Only Ind and B [b]F were highly deviated from other members. Since NHL is located in a remote area where the impacts of industrialisation and urbanisation on PAH contaminations would be minimal, the NHL dendrogram can be interpreted as predominated by biomass combustion.

### 3.6 PCA

PCA is a used multivariate statistical technique and dimensionality-reduction protocol that is commonly employed to decrease the dimensionality of large inter-correlated quantitative dependent parameters. The main principle of PCA is to extract the crucial information from large datasets, and to represent it as a new set of orthogonal variables called PCs. In this study, PCA was performed by organizing the dataset as an *m* × *n* matrix, where *m* is the number of PAHs (*n* = 15) and *n* is the number of sediment samples (n was 73 and 62 for SKL and NHL, respectively). Table S8 exhibits the PC patterns for Varimax rotated components of the SKL PAH dataset. To further explore potential sources of PAHs, a PCA model was computed with five major PCs, each representing 55.0 %, 23.6 %, 9.33 %, 5.48 %, and 2.06 % of the variance, thus accounting for 95.5 % of the total variation in the SKL dataset. PC1 exhibited comparatively high positive loadings of Fluo, Pyr, B [b]F, B [k]F, Ind, and B [g,h,i]P. Since these six compounds were the main compositions of particulate PAHs in the total suspended solids collected on the PSU Campus and downtown Hat-Yai (Tekasakul et al., 2008), the urban emissions (e.g. traffic exhaust and industrial emissions) from Songkhla City contributed approximately 55% of PAH contaminants in SKL sediment layers. Exceedingly strong positive correlation coefficients of Phe and An detected at PC3 suggested that diesel spills from water transportation contribute approximately 9.3%. A previous study found a considerable amount of 11H–B [a]F and 11H–B [b]F in wood smoke emitted during the production of charcoal (Ré-Poppi and Santiago-Silva, 2002). Furthermore, a recent study observed a strong positive correlation between B [a]P and levoglucosan contents in suburban detached-house areas in Helsinki, Finland (Hellén et al., 2017). Since levoglucosan is a well-known biomarker for tracing cellulose in biomass combustion and atmospheric particles (Bae et al., 2012; Simoneit et al., 1999), the strong positive correlation coefficients of 11H–B [a]F, 11H–B [b]F, and B [a]P can be ascribed to biomass combustion with an approximate contribution of 23.6%. A similar PCA was also conducted for the NHL samples, as illustrated in Table S9. Five PCs were successfully classified from the PC matrix, each representing 61.3 %, 20.2 %, 6.96 %, 3.94 %, and 3.39 % of the variance, thus accounting for 95.8 % of the total variance. Exceedingly strong positive correlation coefficients (i.e. *R* > 0.9) for B [a]A, Chry, B [b]F, B [k]F, B [e]P, Ind, D [a,h]A, and B [g,h,i]P were measured at PC1, as illustrated in Table S9. This indicates that biomass burning contributed approximately 61.3% of the PAH contaminants in the NHL sediments. The PCA results were consistent with the other statistical analyses presented in previous sections, underlining the importance of biomass burning as one of the main contributors of PAH contaminants in sediment samples from the NHL.

## 4 Conclusion

The vertical distribution of PAH contents was successfully determined in sediments collected from a pristine natural lake situated in north-eastern Thailand (i.e. NHL) and the largest brackish lake located in southern Thailand (i.e. SKL). The ΣPAH concentrations observed in SKL and NHL sediments were in the range of 19.4–1,218 ng g^−1^ and 94.5–1,112 ng g^−1^, respectively. Bioaccumulation of PAHs into vegetation and micro-biodegradation coupled with co-metabolism are three major factors which may have reduced the PAH contents of NHL surface sediments. On the contrary, the shipping activities were considered as the main contributors of PAHs in subsurface sediments of SKL. Prominently, both lakes have similar proportions of ΣPAHs (3,4) and ΣPAHs (5,6) percentage contributions, of approximately 50 %. These results were remarkably different to those of previous studies underlining the comparatively high proportions of ΣPAHs (3,4) in freshwater sediments collected from lakes greatly affected by human activities. Diagnostic binary ratios indicated that biomass burning was one of the main contributors of PAHs in NHL sediments, while oil spills from boat engines could play a major role in governing PAH contents, particularly in surface sediment layers of the SKL. Phe and An exhibited moderately strong positive correlations with other LMW PAHs, as determined by PPMCC, supporting the idea that oil spills from boat engines are largely responsible for PAH contamination, particularly in surface layer sediments of the SKL. PCA highlighted biomass burning and anthropogenic activities, respectively, as the two main potential sources of PAHs occurring in NHL and SKL sediment samples. In addition, PAH contamination in pristine natural lake sediments in the NHL and SKL can be greatly reduced through policy support of local and national authorities, sharing of successful non-combustion agricultural practices, and international regulations and subsidies for alternative tools to limit agricultural waste burning.

## Declarations

### Author contribution statement

Siwatt Pongpiachan: Conceived and designed the experiments; Analyzed and interpreted the data; Wrote the paper.

Qiyuan Wang; Li Xing; Guohui Li: Conceived and designed the experiments; Performed the experiments.

Danai Tipmanee; Woranuch Deelaman; Chormsri Choochuay; Natthapong Iadtem: Contributed reagents, materials, analysis tools or data.

Yongming Han; Junji Cao: Performed the experiments.

Muhammad Zaffar Hashmi: Analyzed and interpreted the data; Wrote the paper.

### Funding statement

Qiyuan Wang was supported by 10.13039/501100005151Chinese Academy of Sciences Key Project [the Strategic Priority Research Program of Chinese Academy of Sciences (XDB40000000)].

### Data availability statement

Data included in article/supp. material/referenced in article.

### Declaration of interest’s statement

The authors declare no conflict of interest.

### Additional information

No additional information is available for this paper.

## Appendix A Supplementary data

30082022-The Supplementary_Heliyon.docx 30082022-The Supplementary_Heliyon.docx
