# Supplementary material for: Concentrations and source identification of priority polycyclic aromatic hydrocarbons in sediment cores from south and northeast Thailand
Source: Heliyon. 2022 Oct 4;8(10):e10953. doi: 10.1016/j.heliyon.2022.e10953 (PMC9573892; doi:10.1016/j.heliyon.2022.e10953)
Supplement: 30082022-The Supplementary_Heliyon.docx [file mmc1.docx]

**The supplementary information**

**Concentrations and Source Identification of Priority Polycyclic Aromatic Hydrocarbons in Six Lake Sediment Cores from South and Northeast Thailand**

Siwatt Pongpiachan^a*^, Danai Tipmanee^b^, Chomsri Choochuay^c^, Woranuch Deelaman^d^, Natthapong Iadtem^c^, Qiyuan Wang^e*^, Li Xing^f^, Guohui Li^e^, Yongming Han^e^, Muhammad Zaffar Hashmi^g^, Junji Cao^e^, Apichart Leckngam^h^, and Saran Poshyachinda^h^

^a^NIDA Center for Research & Development of Disaster Prevention & Management, School of Social and Environmental Development, National Institute of Development Administration (NIDA),

148 Moo 3, Sereethai Road, Klong-Chan, Bangkapi, Bangkok, 10240, THAILAND

^b^Faculty of Technology and Environment, Prince of Songkla University Phuket Campus 80 M.1 Kathu, Phuket 83120, THAILAND

^c^Faculty of Environmental Management, Prince of Songkla University Hat-Yai Campus, Songkla, 90112, THAILAND

^d^Division of Environmental Science and Technology,Faculty of Science and Technology, Rajamangala University of Technology Phra Nakhon, Bangkok 10800,THAILAND

^e^SKLLQG and Key Lab of Aerosol Chemistry & Physics, Institute of Earth Environment, Chinese Academy of Sciences (IEECAS), Xi’an, 710061, CHINA

^f^School of Geography and Tourism, Shaanxi Normal University, Xi'an, 710119, China; Key Lab of Aerosol Chemistry and Physics, SKLLQG, Institute of Earth Environment, Chinese Academy of Sciences, Xi'an, 710061, CHINA

^g^Department of Chemistry, COMSATS University, Islamabad, PAKISTAN

^h^National Astronomical Research Institute of Thailand (Public Organization), 260 Moo 4, T. Donkaew A. Maerim, Chiang Mai, 50180, THAILAND

^*^Corresponding Authors: Tel: 00 66 2 727 3113; Fax: 00 66 2 732 0276; Email: [pongpiajun@gmail.com](mailto:pongpiajun@gmail.com), [wangqy@ieecas.cn](mailto:wangqy@ieecas.cn)

Table S1. Statistical measurement of accuracy and precision with National Institute of Standards and Technology Standard Reference Material 1941b (NIST-SRM 1941b).

| PAH congener | *n* | Reported Value  (ng g^−1^  dry weight) | Measured Value  (ng g^−1^  dry weight) | % Accuracy |
| --- | --- | --- | --- | --- |
| Phe | 8 | 406±44.0 | 464±16.0 | 114 |
| Fluo | 8 | 651±50.0 | 721±45.0 | 111 |
| Pyr | 8 | 581±39.0 | 538±34.0 | 93 |
| B[a]A | 8 | 335±25.0 | 289±26.0 | 86 |
| Chry | 8 | 291±31.0 | 336±25.0 | 115 |
| B[b]F | 8 | 453±21.0 | 480±25.0 | 106 |
| B[k]F | 8 | 225±18.0 | 229±19.0 | 102 |
| B[e]P | 8 | 325±25.0 | 321±10.0 | 99 |
| Ind | 8 | 341±57.0 | 291±14.0 | 85 |
| B[g,h,i]P | 8 | 307±45.0 | 267±17.0 | 87 |

Table S2. Statistical descriptions of An/(Phe+An) collected at SKL and NHL in comparison with values of a previous study (Yunker et al., 2011)

|  |  |  |  |  |  |  |  |  |  |  |  |  |  |  |  |  |  |  | **Petroleum** | **Liquid fossil fuel combustion/**  **Mixed sources** | **Grass/wood (biomass)**  **/coal combustion** |
| --- | --- | --- | --- | --- | --- | --- | --- | --- | --- | --- | --- | --- | --- | --- | --- | --- | --- | --- | --- | --- | --- |
| **Depth** | **SKL1** | **SKL2** | **SKL3** | **NHL1** | **NHL2** | **NHL3** | **SKL1** | **SKL2** | **SKL3** | **NHL1** | **NHL2** | **NHL3** | **SKL1** | **SKL2** | **SKL3** | **NHL1** | **NHL2** | **NHL3** |  |  |  |
| **[m]** | **An** | **An** | **An** | **An** | **An** | **An** | **Phe** | **Phe** | **Phe** | **Phe** | **Phe** | **Phe** | **An/(Phe+An)** | | | | | | | | |
| 0 | 1.97 | 1.27 | 1.31 | 8.24 | 6.49 | 3.86 | 11.89 | 6.34 | 7.53 | 52.31 | 37.12 | 26.98 | 0.14 | 0.17 | 0.15 | 0.14 | 0.15 | 0.13 | <0.10 | >0.10 | >0.10 |
| 2 | 2.61 | 1.13 | 1.28 | 6.4 | 6.89 | 3.72 | 15.14 | 5.56 | 5.85 | 42.05 | 38.12 | 26.13 | 0.15 | 0.17 | 0.18 | 0.13 | 0.15 | 0.12 |  | | |
| 4 | 2.9 | 0.84 | 1.34 | 7.71 | 6.43 | 5 | 18.2 | 5.13 | 5.59 | 44.73 | 37.86 | 35 | 0.14 | 0.14 | 0.19 | 0.15 | 0.15 | 0.13 |  |  |  |
| 6 | 2.75 | 0.93 | 1.45 | 7.92 | 6.56 | 3.15 | 16.54 | 7.19 | 5.97 | 52.55 | 36.31 | 27.07 | 0.14 | 0.11 | 0.20 | 0.13 | 0.15 | 0.10 |  |  |  |
| 8 | 4.68 | 1.35 | 1.19 | 7.67 | 5.2 | 2.84 | 27.09 | 6.33 | 5.12 | 41.11 | 19.12 | 21.84 | 0.15 | 0.18 | 0.19 | 0.16 | 0.21 | 0.12 |  |  |  |
| 10 | 2.93 | 0.73 | 0.97 | 8.29 | 5.41 | 2.29 | 18.06 | 5.72 | 12.24 | 46.56 | 22.74 | 17.54 | 0.14 | 0.11 | 0.07 | 0.15 | 0.19 | 0.12 |  |  |  |
| 12 | 2.25 | 0.82 | 0.67 | 7.02 | 5.77 | 2.75 | 13.7 | 5.33 | 10.8 | 42.16 | 22.92 | 18.95 | 0.14 | 0.13 | 0.06 | 0.14 | 0.20 | 0.13 |  |  |  |
| 14 | 3.22 | 0.64 | 0.72 | 4.77 | 6.33 | 3.24 | 18.14 | 4.19 | 4.99 | 26.3 | 26.72 | 20.58 | 0.15 | 0.13 | 0.13 | 0.15 | 0.19 | 0.14 |  |  |  |
| 16 | 3.97 | 1.35 | 0.76 | 4.27 | 5.72 | 1.36 | 24.22 | 4.93 | 6.02 | 30.69 | 34.35 | 22.04 | 0.14 | 0.21 | 0.11 | 0.12 | 0.14 | 0.06 |  |  |  |
| 18 | 5.5 | 0.39 | 0.53 | 4.4 | 6.73 | 2.47 | 31.3 | 2.77 | 4.8 | 20.57 | 26.05 | 17.65 | 0.15 | 0.12 | 0.10 | 0.18 | 0.21 | 0.12 |  |  |  |
| 20 | 7.19 | 0.26 | 0.57 | 3.69 | 3.93 | 2.35 | 43.51 | 3.11 | 9.32 | 19.06 | 17.95 | 15.28 | 0.14 | 0.08 | 0.06 | 0.16 | 0.18 | 0.13 |  |  |  |
| 22 | 5.99 | 0.21 | 0.4 | 4.18 | 4.1 | 2.61 | 21.27 | 2.08 | 6.54 | 16.82 | 17.81 | 14.89 | 0.22 | 0.09 | 0.06 | 0.20 | 0.19 | 0.15 |  |  |  |
| 24 | 7.39 | 0.23 | 0.27 | 3.47 | 3.2 | 3.1 | 25.65 | 3.66 | 7.18 | 16.42 | 16.97 | 13.06 | 0.22 | 0.06 | 0.04 | 0.17 | 0.16 | 0.19 |  |  |  |
| 26 | 6.32 | 0.19 | 0.31 | 3.47 | 2.87 | 5.75 | 30.11 | 1.97 | 6.76 | 13.26 | 11.98 | 21.09 | 0.17 | 0.09 | 0.04 | 0.21 | 0.19 | 0.21 |  |  |  |
| 28 | 7.97 | 0.21 | 0.17 | 3.21 | 1.75 | 5.23 | 63.7 | 3.78 | 5.44 | 12.5 | 15.03 | 18.13 | 0.11 | 0.05 | 0.03 | 0.20 | 0.10 | 0.22 |  |  |  |
| 30 | 8.61 | 0.19 | 0.32 | 3.24 | 1.54 | 6.75 | 49.9 | 1.84 | 6.19 | 11.43 | 17.22 | 17.56 | 0.15 | 0.09 | 0.05 | 0.22 | 0.08 | 0.28 |  |  |  |
| 32 | 5.38 |  | 0.34 | 2.94 |  | 9.75 | 32.12 |  | 5.35 | 10.24 |  | 23.06 | 0.14 |  | 0.06 | 0.22 |  | 0.30 |  |  |  |
| 34 | 4.96 |  | 0.28 | 3.17 |  | 13.32 | 17.75 |  | 6.89 | 12.74 |  | 26.46 | 0.22 |  | 0.04 | 0.20 |  | 0.33 |  |  |  |
| 36 | 4.4 |  | 0.23 | 3.7 |  | 7.52 | 19.99 |  | 5.08 | 14.89 |  | 19.21 | 0.18 |  | 0.04 | 0.20 |  | 0.28 |  |  |  |
| 38 | 4.66 |  | 0.26 | 3.58 |  | 9.23 | 20.24 |  | 3.86 | 10.33 |  | 19.9 | 0.19 |  | 0.06 | 0.26 |  | 0.32 |  |  |  |
| 40 | 5.45 |  | 0.24 | 4.84 |  |  | 19.3 |  | 4.08 | 13.84 |  |  | 0.22 |  | 0.06 | 0.26 |  |  |  |  |  |
| 42 | 4.3 |  | 0.26 | 4.2 |  |  | 18.12 |  | 5.62 | 18.26 |  |  | 0.19 |  | 0.04 | 0.19 |  |  |  |  |  |
| 44 | 5.34 |  | 0.3 | 4.59 |  |  | 16.03 |  | 6.23 | 17.9 |  |  | 0.25 |  | 0.05 | 0.20 |  |  |  |  |  |
| 46 | 5.73 |  | 0.24 | 4.92 |  |  | 24.19 |  | 5.21 | 15.49 |  |  | 0.19 |  | 0.04 | 0.24 |  |  |  |  |  |
| 48 | 4.54 |  | 0.21 | 5.78 |  |  | 22.25 |  | 2.37 | 21.23 |  |  | 0.17 |  | 0.08 | 0.21 |  |  |  |  |  |
| 50 | 2.93 |  | 0.23 | 6.02 |  |  | 16.81 |  | 6.05 | 24.14 |  |  | 0.15 |  | 0.04 | 0.20 |  |  |  |  |  |
| 52 | 2.97 |  | 0.24 |  |  |  | 11.91 |  | 6.58 |  |  |  | 0.20 |  | 0.04 |  |  |  |  |  |  |
| 54 | 3.3 |  | 0.33 |  |  |  | 14.03 |  | 5.14 |  |  |  | 0.19 |  | 0.06 |  |  |  |  |  |  |
| 56 | 2.01 |  |  |  |  |  | 12.85 |  |  |  |  |  | 0.14 |  |  |  |  |  |  |  |  |

Table S3. Statistical descriptions of Fluo/(Fluo+Pyr) collected at SKL and NHL in comparison with values of a previous study (Yunker et al., 2011)

|  |  |  |  |  |  |  |  |  |  |  |  |  |  |  |  |  |  |  | **Petroleum** | **Liquid fossil fuel combustion/**  **Mixed sources** | **Grass/wood (biomass)**  **/coal combustion** |
| --- | --- | --- | --- | --- | --- | --- | --- | --- | --- | --- | --- | --- | --- | --- | --- | --- | --- | --- | --- | --- | --- |
| **Depth** | **SKL1** | **SKL2** | **SKL3** | **NHL1** | **NHL2** | **NHL3** | **SKL1** | **SKL2** | **SKL3** | **NHL1** | **NHL2** | **NHL3** | **SKL1** | **SKL2** | **SKL3** | **NHL1** | **NHL2** | **NHL3** |  |  |  |
| **[m]** | **Fluo** | **Fluo** | **Fluo** | **Fluo** | **Fluo** | **Fluo** | **Pyr** | **Pyr** | **Pyr** | **Pyr** | **Pyr** | **Pyr** | **Fluo/(Fluo+Pyr)** | | | | | | | | |
| 0 | 13.7 | 11.2 | 19.53 | 42.8 | 36.8 | 27.1 | 17.0 | 16.1 | 16.1 | 37.0 | 36.79 | 24.8 | 0.45 | 0.41 | 0.55 | 0.54 | 0.50 | 0.52 | <0.40 | 0.40-0.50 | >0.50 |
| 2 | 21.2 | 14.3 | 18.18 | 38.6 | 41.1 | 23.4 | 37.0 | 23.8 | 18.9 | 34.3 | 42.7 | 20.7 | 0.36 | 0.37 | 0.49 | 0.53 | 0.49 | 0.53 |  | | |
| 4 | 25.1 | 12.8 | 17.76 | 41.7 | 36.0 | 29.6 | 29.5 | 18.1 | 18.7 | 34.6 | 37.28 | 26.3 | 0.46 | 0.41 | 0.49 | 0.55 | 0.49 | 0.53 |  |  |  |
| 6 | 21.7 | 15.6 | 15.51 | 50.1 | 37.6 | 24.3 | 25.2 | 16.7 | 16.2 | 42.0 | 43.29 | 20.3 | 0.46 | 0.48 | 0.49 | 0.54 | 0.46 | 0.55 |  |  |  |
| 8 | 65.9 | 17.1 | 13.08 | 40.9 | 36.7 | 19.5 | 197 | 13.8 | 13.4 | 32.9 | 43.45 | 15.5 | 0.25 | 0.55 | 0.49 | 0.55 | 0.46 | 0.56 |  |  |  |
| 10 | 33.9 | 10.0 | 11.68 | 46.8 | 43.9 | 15.1 | 32.7 | 6.2 | 12.2 | 41.6 | 42.5 | 12.6 | 0.51 | 0.62 | 0.49 | 0.53 | 0.51 | 0.54 |  |  |  |
| 12 | 32.5 | 8.9 | 9.6 | 44.5 | 44.0 | 16.4 | 27.0 | 4.5 | 8.93 | 40.3 | 42.02 | 15.0 | 0.55 | 0.66 | 0.52 | 0.52 | 0.51 | 0.52 |  |  |  |
| 14 | 37.7 | 9.0 | 8.99 | 39.3 | 43.5 | 16.7 | 35.0 | 4.7 | 8.31 | 35.8 | 43.28 | 14.5 | 0.52 | 0.66 | 0.52 | 0.52 | 0.50 | 0.54 |  |  |  |
| 16 | 43.4 | 14.4 | 8.3 | 35.5 | 45.8 | 18.0 | 42.7 | 7.2 | 7.75 | 34.1 | 45.25 | 14.2 | 0.50 | 0.67 | 0.52 | 0.51 | 0.50 | 0.56 |  |  |  |
| 18 | 38.9 | 3.6 | 6.05 | 33.8 | 41.2 | 16.5 | 49.2 | 2.3 | 5.57 | 29.5 | 38.38 | 10.7 | 0.44 | 0.61 | 0.52 | 0.53 | 0.52 | 0.61 |  |  |  |
| 20 | 33.4 | 2.1 | 5.63 | 28.3 | 26.0 | 20.2 | 41.7 | 2.8 | 5.51 | 25.0 | 22.57 | 10.8 | 0.44 | 0.44 | 0.51 | 0.53 | 0.54 | 0.65 |  |  |  |
| 22 | 37.1 | 1.5 | 4.28 | 30.9 | 28.0 | 25.3 | 47.4 | 1.2 | 4.08 | 30.8 | 22.54 | 13.7 | 0.44 | 0.55 | 0.51 | 0.50 | 0.55 | 0.65 |  |  |  |
| 24 | 33.7 | 1.8 | 2.48 | 25.5 | 21.5 | 29.6 | 41.5 | 1.5 | 2.68 | 26.5 | 18.92 | 16.9 | 0.45 | 0.54 | 0.48 | 0.49 | 0.53 | 0.64 |  |  |  |
| 26 | 24.2 | 1.8 | 2.49 | 25.0 | 18.7 | 51.7 | 29.0 | 2.7 | 2.38 | 25.2 | 17.1 | 34.1 | 0.45 | 0.40 | 0.51 | 0.50 | 0.52 | 0.60 |  |  |  |
| 28 | 31.9 | 2.7 | 1.95 | 21.5 | 12.4 | 29.1 | 40.8 | 4.8 | 2.21 | 25.0 | 12.55 | 21.6 | 0.44 | 0.36 | 0.47 | 0.46 | 0.50 | 0.57 |  |  |  |
| 30 | 38.1 | 1.8 | 2.26 | 19.9 | 12.5 | 39.4 | 46.9 | 3.3 | 2.20 | 24.6 | 12.94 | 34.7 | 0.45 | 0.36 | 0.51 | 0.45 | 0.49 | 0.53 |  |  |  |
| 32 | 23.2 |  | 2.15 | 17.3 |  | 56.9 | 27.3 |  | 2.18 | 22.0 |  | 51.4 | 0.46 |  | 0.50 | 0.44 |  | 0.52 |  |  |  |
| 34 | 24.5 |  | 2.36 | 18.2 |  | 68.7 | 27.8 |  | 2.18 | 22.8 |  | 55.3 | 0.47 |  | 0.52 | 0.44 |  | 0.55 |  |  |  |
| 36 | 26.2 |  | 1.99 | 20.7 |  | 52.6 | 29.8 |  | 2.14 | 26.0 |  | 37.1 | 0.47 |  | 0.48 | 0.44 |  | 0.59 |  |  |  |
| 38 | 31.7 |  | 2.06 | 20.2 |  | 55.6 | 35.2 |  | 2.00 | 25.9 |  | 40.9 | 0.47 |  | 0.51 | 0.44 |  | 0.58 |  |  |  |
| 40 | 32.5 |  | 1.61 | 29.5 |  |  | 40.2 |  | 1.62 | 40.9 |  |  | 0.45 |  | 0.50 | 0.42 |  |  |  |  |  |
| 42 | 36.0 |  | 1.84 | 26.0 |  |  | 43.5 |  | 1.70 | 36.8 |  |  | 0.45 |  | 0.52 | 0.41 |  |  |  |  |  |
| 44 | 35.9 |  | 1.98 | 27.1 |  |  | 47.0 |  | 1.87 | 40.5 |  |  | 0.43 |  | 0.51 | 0.40 |  |  |  |  |  |
| 46 | 36.9 |  | 1.92 | 30.8 |  |  | 66.7 |  | 2.01 | 47.8 |  |  | 0.36 |  | 0.49 | 0.39 |  |  |  |  |  |
| 48 | 34.3 |  | 1.5 | 35.2 |  |  | 50.0 |  | 1.60 | 62.1 |  |  | 0.41 |  | 0.48 | 0.36 |  |  |  |  |  |
| 50 | 15.9 |  | 2.45 | 38.9 |  |  | 24.4 |  | 2.53 | 64.7 |  |  | 0.39 |  | 0.49 | 0.38 |  |  |  |  |  |
| 52 | 18.4 |  | 2.08 |  |  |  | 23.8 |  | 1.98 |  |  |  | 0.44 |  | 0.51 |  |  |  |  |  |  |
| 54 | 20.1 |  | 2.04 |  |  |  | 26.2 |  | 1.96 |  |  |  | 0.43 |  | 0.51 |  |  |  |  |  |  |
| 56 | 12.0 |  |  |  |  |  | 14.8 |  |  |  |  |  | 0.45 |  |  |  |  |  |  |  |  |

Table S4. Statistical descriptions of B[a]A/(B[a]A+Chry) collected at SKL and NHL in comparison with values of a previous study (Yunker et al., 2011)

|  |  |  |  |  |  |  |  |  |  |  |  |  |  |  |  |  |  |  | **Petroleum** | **Liquid fossil fuel combustion/**  **Mixed sources** | **Grass/wood (biomass)**  **/coal combustion** |
| --- | --- | --- | --- | --- | --- | --- | --- | --- | --- | --- | --- | --- | --- | --- | --- | --- | --- | --- | --- | --- | --- |
| **Depth** | **SKL1** | **SKL2** | **SKL3** | **NHL1** | **NHL2** | **NHL3** | **SKL1** | **SKL2** | **SKL3** | **NHL1** | **NHL2** | **NHL3** | **SKL1** | **SKL2** | **SKL3** | **NHL1** | **NHL2** | **NHL3** |  |  |  |
| **[m]** | **B[a]A** | **B[a]A** | **B[a]A** | **B[a]A** | **B[a]A** | **B[a]A** | **Chry** | **Chry** | **Chry** | **Chry** | **Chry** | **Chry** | **B[a]A/(B[a]A+Chry)** | | | | | | | | |
| 0 | 2.7 | 3.8 | 4.01 | 7.0 | 6.8 | 5.9 | 3.9 | 6.4 | 9.2 | 13.1 | 13.45 | 13.3 | 0.41 | 0.37 | 0.30 | 0.35 | 0.33 | 0.31 | <0.20 | 0.20-0.35 | >0.35 |
| 2 | 3.2 | 5.9 | 4.73 | 6.1 | 8.1 | 3.5 | 4.7 | 8.9 | 9.7 | 13.1 | 16.71 | 4.4 | 0.40 | 0.40 | 0.33 | 0.32 | 0.33 | 0.44 |  | | |
| 4 | 5.1 | 3.2 | 5.78 | 8.0 | 7.2 | 3.6 | 7.5 | 7.3 | 10.9 | 14.8 | 13.47 | 4.0 | 0.40 | 0.30 | 0.35 | 0.35 | 0.35 | 0.47 |  |  |  |
| 6 | 3.6 | 2.8 | 5 | 8.6 | 8.8 | 3.1 | 5.7 | 7.9 | 9.4 | 17.8 | 18.98 | 2.3 | 0.38 | 0.26 | 0.35 | 0.33 | 0.32 | 0.58 |  |  |  |
| 8 | 3.5 | 3.2 | 4.71 | 7.3 | 8.1 | 2.4 | 7 | 8.6 | 7.9 | 15.2 | 20.19 | 5.8 | 0.32 | 0.27 | 0.37 | 0.32 | 0.29 | 0.29 |  |  |  |
| 10 | 6.2 | 1.9 | 3.38 | 8.7 | 8.2 | 1.9 | 15.4 | 5.5 | 6.2 | 17.8 | 16.18 | 4.3 | 0.29 | 0.25 | 0.35 | 0.33 | 0.34 | 0.31 |  |  |  |
| 12 | 5.3 | 1.9 | 2.53 | 10.8 | 9.2 | 2.9 | 18.6 | 5.1 | 5.05 | 21.5 | 17.01 | 5.5 | 0.22 | 0.27 | 0.33 | 0.33 | 0.35 | 0.34 |  |  |  |
| 14 | 5.2 | 2.1 | 2.57 | 9.2 | 9.4 | 3.1 | 33.4 | 5.1 | 5.69 | 22.2 | 17.75 | 2.4 | 0.13 | 0.29 | 0.31 | 0.29 | 0.35 | 0.57 |  |  |  |
| 16 | 4.8 | 4.3 | 2.41 | 6.0 | 9.6 | 2.4 | 52.9 | 7.5 | 5.33 | 15.8 | 20.19 | 7.2 | 0.08 | 0.36 | 0.31 | 0.28 | 0.32 | 0.25 |  |  |  |
| 18 | 10.7 | 1.0 | 1.63 | 8.0 | 10.1 | 2.7 | 45.9 | 2.2 | 3.86 | 18.3 | 20.58 | 7.3 | 0.19 | 0.31 | 0.30 | 0.30 | 0.33 | 0.27 |  |  |  |
| 20 | 3.6 | 0.3 | 1.09 | 5.5 | 4.5 | 2.5 | 94.8 | 0.9 | 3.20 | 12.8 | 13.01 | 3.4 | 0.04 | 0.29 | 0.25 | 0.30 | 0.25 | 0.43 |  |  |  |
| 22 | 7.7 | 0.4 | 0.81 | 8.9 | 6.8 | 2.8 | 121.3 | 1.2 | 2.60 | 16.7 | 15.54 | 8.6 | 0.06 | 0.25 | 0.24 | 0.35 | 0.31 | 0.25 |  |  |  |
| 24 | 5.6 | 0.5 | 0.38 | 9.1 | 5.0 | 3.3 | 125.3 | 1.2 | 1.69 | 15.5 | 14.61 | 8.7 | 0.04 | 0.28 | 0.18 | 0.37 | 0.26 | 0.28 |  |  |  |
| 26 | 5.8 | 0.3 | 0.36 | 7.3 | 5.0 | 6.0 | 81.5 | 0.8 | 1.76 | 14.2 | 14.72 | 14.8 | 0.07 | 0.29 | 0.17 | 0.34 | 0.25 | 0.29 |  |  |  |
| 28 | 5.2 | 0.4 | 0.3 | 8.7 | 3.8 | 3.9 | 96.4 | 1.1 | 1.78 | 16.9 | 11.91 | 8.0 | 0.05 | 0.25 | 0.14 | 0.34 | 0.24 | 0.33 |  |  |  |
| 30 | 8.9 | 0.4 | 0.31 | 8.8 | 4.5 | 10.5 | 89.5 | 1.0 | 1.09 | 18.0 | 12.31 | 19.5 | 0.09 | 0.29 | 0.22 | 0.33 | 0.27 | 0.35 |  |  |  |
| 32 | 4.6 |  | 0.35 | 9.1 |  | 18.2 | 68.2 |  | 1.26 | 20.3 |  | 33.6 | 0.06 |  | 0.22 | 0.31 |  | 0.35 |  |  |  |
| 34 | 6.5 |  | 0.33 | 8.9 |  | 24.7 | 74.7 |  | 1.40 | 21.7 |  | 41.9 | 0.08 |  | 0.19 | 0.29 |  | 0.37 |  |  |  |
| 36 | 8.7 |  | 0.33 | 11.8 |  | 17.8 | 71.7 |  | 1.63 | 30.5 |  | 41.4 | 0.11 |  | 0.17 | 0.28 |  | 0.30 |  |  |  |
| 38 | 9.5 |  | 0.34 | 12.0 |  | 21.5 | 75.4 |  | 1.54 | 31.0 |  | 44.8 | 0.11 |  | 0.18 | 0.28 |  | 0.32 |  |  |  |
| 40 | 10.9 |  | 0.24 | 22.4 |  |  | 54.6 |  | 0.99 | 63.5 |  |  | 0.17 |  | 0.20 | 0.26 |  |  |  |  |  |
| 42 | 11.9 |  | 0.3 | 17.5 |  |  | 37.7 |  | 0.97 | 52.6 |  |  | 0.24 |  | 0.24 | 0.25 |  |  |  |  |  |
| 44 | 15.8 |  | 0.31 | 18.7 |  |  | 37.5 |  | 0.99 | 54.8 |  |  | 0.30 |  | 0.24 | 0.25 |  |  |  |  |  |
| 46 | 7.1 |  | 0.3 | 25.0 |  |  | 20.2 |  | 1.01 | 73.2 |  |  | 0.26 |  | 0.23 | 0.25 |  |  |  |  |  |
| 48 | 8.5 |  | 0.32 | 35.0 |  |  | 16.1 |  | 1.17 | 99.7 |  |  | 0.35 |  | 0.21 | 0.26 |  |  |  |  |  |
| 50 | 3.6 |  | 0.42 | 32.2 |  |  | 11.0 |  | 1.25 | 98.2 |  |  | 0.24 |  | 0.25 | 0.25 |  |  |  |  |  |
| 52 | 15.4 |  | 0.25 |  |  |  | 11.6 |  | 0.90 |  |  |  | 0.57 |  | 0.22 |  |  |  |  |  |  |
| 54 | 8.1 |  | 0.34 |  |  |  | 22.3 |  | 1.02 |  |  |  | 0.27 |  | 0.25 |  |  |  |  |  |  |
| 56 | 6.5 |  |  |  |  |  | 14.7 |  |  |  |  |  | 0.31 |  |  |  |  |  |  |  |  |

Table S5. Statistical descriptions of B[b+j+k]F/( B[b+j+k]F+B[e]P) collected at SKL and NHL in comparison with values of a previous study (Yunker et al., 2011)

|  |  |  |  |  |  |  |  |  |  |  |  |  |  |  |  |  |  |  | **Petroleum** | **Liquid fossil fuel combustion/**  **Mixed sources** | **Grass/wood (biomass)**  **/coal combustion** |
| --- | --- | --- | --- | --- | --- | --- | --- | --- | --- | --- | --- | --- | --- | --- | --- | --- | --- | --- | --- | --- | --- |
| **Depth** | **SKL1** | **SKL2** | **SKL3** | **NHL1** | **NHL2** | **NHL3** | **SKL1** | **SKL2** | **SKL3** | **NHL1** | **NHL2** | **NHL3** | **SKL1** | **SKL2** | **SKL3** | **NHL1** | **NHL2** | **NHL3** |  |  |  |
| **[m]** | **B[b+j+k]F** | **B[b+j+k]F** | **B[b+j+k]F** | **B[b+j+k]F** | **B[b+j+k]F** | **B[b+j+k]F** | **B[e]P** | **B[e]P** | **B[e]P** | **B[e]P** | **B[e]P** | **B[e]P** | **B[b+j+k]F/(B[b+j+k]F+B[e]P)** | | | | | | | | |
| 0 | 20.3 | 13.3 | 14.14 | 37.7 | 38.0 | 22.9 | 6.58 | 5.95 | 5.76 | 12.8 | 12.7 | 8.13 | 0.76 | 0.69 | 0.71 | 0.75 | 0.75 | 0.74 | <0.50 | 0.50-0.70 | >0.70 |
| 2 | 27.2 | 19.7 | 16.31 | 38.7 | 36.3 | 15.5 | 10.19 | 6.97 | 6.51 | 13.6 | 12.84 | 7.53 | 0.73 | 0.74 | 0.71 | 0.74 | 0.74 | 0.67 |  | | |
| 4 | 23.4 | 12.7 | 17.71 | 47.7 | 29.1 | 16.8 | 7.43 | 5.04 | 6.54 | 15.8 | 10.54 | 7.80 | 0.76 | 0.72 | 0.73 | 0.75 | 0.73 | 0.68 |  |  |  |
| 6 | 27.9 | 8.6 | 18.03 | 44.3 | 41.4 | 12.2 | 8.24 | 3.49 | 6.47 | 15.7 | 12.53 | 5.64 | 0.77 | 0.71 | 0.74 | 0.74 | 0.77 | 0.68 |  |  |  |
| 8 | 85.1 | 9.5 | 14.55 | 45.8 | 41.4 | 12.4 | 7.12 | 4.42 | 5.20 | 15.0 | 10.8 | 5.26 | 0.92 | 0.68 | 0.74 | 0.75 | 0.79 | 0.70 |  |  |  |
| 10 | 31.2 | 10.4 | 9.96 | 45.3 | 41.1 | 10.0 | 9.37 | 4.49 | 3.41 | 14.9 | 10.44 | 4.65 | 0.77 | 0.70 | 0.74 | 0.75 | 0.80 | 0.68 |  |  |  |
| 12 | 14.4 | 9.3 | 10.57 | 53.8 | 49.1 | 9.5 | 7.25 | 3.63 | 3.50 | 17.1 | 12.37 | 4.68 | 0.67 | 0.72 | 0.75 | 0.76 | 0.80 | 0.67 |  |  |  |
| 14 | 22.9 | 9.3 | 8.74 | 51.8 | 53.8 | 13.2 | 6.63 | 3.39 | 2.90 | 15.8 | 16.68 | 4.71 | 0.78 | 0.73 | 0.75 | 0.77 | 0.76 | 0.74 |  |  |  |
| 16 | 21.5 | 19.1 | 8.86 | 46.1 | 61.0 | 16.0 | 5.37 | 6.22 | 2.71 | 15.8 | 16.88 | 6.32 | 0.80 | 0.75 | 0.77 | 0.74 | 0.78 | 0.72 |  |  |  |
| 18 | 27.9 | 5.0 | 6.86 | 44.8 | 74.0 | 15.6 | 4.23 | 1.57 | 2.00 | 13.5 | 22.34 | 4.86 | 0.87 | 0.76 | 0.77 | 0.77 | 0.77 | 0.76 |  |  |  |
| 20 | 9.6 | 2.2 | 4.92 | 38.5 | 41.8 | 16.9 | 1.35 | 0.74 | 1.56 | 10.3 | 13.46 | 4.61 | 0.88 | 0.75 | 0.76 | 0.79 | 0.76 | 0.79 |  |  |  |
| 22 | 12.2 | 2.0 | 4.88 | 44.7 | 57.5 | 14.3 | 2.20 | 0.50 | 1.24 | 12.0 | 16.74 | 4.09 | 0.85 | 0.80 | 0.80 | 0.79 | 0.77 | 0.78 |  |  |  |
| 24 | 15.7 | 2.1 | 2.69 | 46.3 | 48.3 | 16.5 | 1.92 | 0.55 | 0.69 | 12.7 | 15.63 | 4.76 | 0.89 | 0.79 | 0.80 | 0.79 | 0.76 | 0.78 |  |  |  |
| 26 | 8.1 | 1.9 | 3.81 | 41.5 | 45.1 | 29.8 | 1.05 | 0.71 | 0.84 | 11.8 | 15.46 | 7.86 | 0.88 | 0.73 | 0.82 | 0.78 | 0.74 | 0.79 |  |  |  |
| 28 | 3.6 | 1.9 | 2.88 | 62.5 | 43.4 | 57.0 | 1.20 | 0.92 | 0.59 | 15.8 | 13.39 | 13.34 | 0.75 | 0.67 | 0.83 | 0.80 | 0.76 | 0.81 |  |  |  |
| 30 | 5.1 | 2.0 | 2.42 | 68.1 | 40.6 | 61.6 | 1.48 | 0.74 | 0.51 | 17.7 | 12.79 | 16.83 | 0.78 | 0.73 | 0.83 | 0.79 | 0.76 | 0.79 |  |  |  |
| 32 | 3.8 |  | 2.36 | 74.7 |  | 93.0 | 1.15 |  | 0.50 | 20.5 |  | 26.63 | 0.77 |  | 0.83 | 0.78 |  | 0.78 |  |  |  |
| 34 | 3.3 |  | 2.34 | 95.7 |  | 127.6 | 0.83 |  | 0.47 | 24.8 |  | 34.05 | 0.80 |  | 0.83 | 0.79 |  | 0.79 |  |  |  |
| 36 | 5.0 |  | 2.78 | 123.6 |  | 143.5 | 0.94 |  | 0.61 | 31.6 |  | 35.70 | 0.84 |  | 0.82 | 0.80 |  | 0.80 |  |  |  |
| 38 | 6.1 |  | 2.48 | 135.4 |  | 165.6 | 1.38 |  | 0.55 | 32.6 |  | 39.61 | 0.82 |  | 0.82 | 0.81 |  | 0.81 |  |  |  |
| 40 | 10.8 |  | 2.22 | 229.1 |  |  | 2.96 |  | 0.47 | 54.1 |  |  | 0.78 |  | 0.83 | 0.81 |  |  |  |  |  |
| 42 | 10.8 |  | 2.15 | 215.4 |  |  | 2.66 |  | 0.45 | 51.8 |  |  | 0.80 |  | 0.83 | 0.81 |  |  |  |  |  |
| 44 | 15.5 |  | 2.35 | 215.5 |  |  | 3.37 |  | 0.43 | 51.7 |  |  | 0.82 |  | 0.85 | 0.81 |  |  |  |  |  |
| 46 | 21.4 |  | 2.34 | 311.1 |  |  | 4.84 |  | 0.51 | 73.5 |  |  | 0.82 |  | 0.82 | 0.81 |  |  |  |  |  |
| 48 | 26.4 |  | 2.31 | 425.6 |  |  | 7.86 |  | 0.47 | 93.7 |  |  | 0.77 |  | 0.83 | 0.82 |  |  |  |  |  |
| 50 | 14.9 |  | 2.01 | 398.7 |  |  | 3.04 |  | 0.44 | 84.5 |  |  | 0.83 |  | 0.82 | 0.83 |  |  |  |  |  |
| 52 | 15.1 |  | 1.73 |  |  |  | 2.63 |  | 0.47 |  |  |  | 0.85 |  | 0.79 |  |  |  |  |  |  |
| 54 | 11.3 |  | 2.03 |  |  |  | 4.90 |  | 0.43 |  |  |  | 0.70 |  | 0.83 |  |  |  |  |  |  |
| 56 | 6.1 |  |  |  |  |  | 1.68 |  |  |  |  |  | 0.78 |  |  |  |  |  |  |  |  |

Table S6. PPMCC of PAH congeners in lake sediments collected at SKL. Any values that higher than 0.5 will be highlighted as bold.

|  | Phe | An | Fluo | Pyr | 11H-B[a]F | 11H-B[b]F | B[a]A | Chry | B[b]F | B[k]F | B[e]P | B[a]P | Ind | D[a,h]A | B[g,h,i]P |
| --- | --- | --- | --- | --- | --- | --- | --- | --- | --- | --- | --- | --- | --- | --- | --- |
| Phe | 1 |  |  |  |  |  |  |  |  |  |  |  |  |  |  |
| An | **.915^**^** | 1 |  |  |  |  |  |  |  |  |  |  |  |  |  |
| Fluo | **.739^**^** | **.845^**^** | 1 |  |  |  |  |  |  |  |  |  |  |  |  |
| Pyr | **.585^**^** | **.652^**^** | **.866^**^** | 1 |  |  |  |  |  |  |  |  |  |  |  |
| 11H-B[a]F | .499^**^ | **.715^**^** | **.678^**^** | .422^**^ | 1 |  |  |  |  |  |  |  |  |  |  |
| 11H-B[b]F | **.590^**^** | **.775^**^** | **.705^**^** | .475^**^ | **.921^**^** | 1 |  |  |  |  |  |  |  |  |  |
| B[a]A | **.505^**^** | **.710^**^** | **.720^**^** | .483^**^ | **.879^**^** | **.864^**^** | 1 |  |  |  |  |  |  |  |  |
| Chry | **.784^**^** | **.879^**^** | **.637^**^** | .393^**^ | **.586^**^** | **.614^**^** | **.532^**^** | 1 |  |  |  |  |  |  |  |
| B[b]F | .266^*^ | .315^**^ | **.690^**^** | **.818^**^** | .162 | .205 | .309^**^ | .013 | 1 |  |  |  |  |  |  |
| B[k]F | .292^*^ | .392^**^ | **.706^**^** | **.887^**^** | .354^**^ | .369^**^ | .423^**^ | .096 | **.897^**^** | 1 |  |  |  |  |  |
| B[e]P | .096 | .166 | **.505^**^** | .406^**^ | .232^*^ | .231^*^ | .343^**^ | -.101 | **.753^**^** | **.585^**^** | 1 |  |  |  |  |
| B[a]P | **.601^**^** | **.780^**^** | **.806^**^** | **.739^**^** | **.743^**^** | **.845^**^** | **.704^**^** | **.537^**^** | **.538^**^** | **.674^**^** | .382^**^ | 1 |  |  |  |
| Ind | .221 | .237^*^ | **.579^**^** | **.851^**^** | .061 | .075 | .168 | -.024 | **.899^**^** | **.911^**^** | .499^**^ | .441^**^ | 1 |  |  |
| D[a,h]A | .359^**^ | .483^**^ | **.607^**^** | .409^**^ | .447^**^ | .463^**^ | **.532^**^** | .379^**^ | **.515^**^** | .463^**^ | **.687^**^** | **.520^**^** | .332^**^ | 1 |  |
| B[g,h,i]P | .167 | .150 | .457^**^ | **.807^**^** | -.070 | -.044 | .009 | -.052 | **.783^**^** | **.829^**^** | .284^*^ | .340^**^ | **.961^**^** | .152 | 1 |

| **. Correlation is significant at the 0.01 level (2-tailed). |
| --- |
| *. Correlation is significant at the 0.05 level (2-tailed). |

Where; phenanthrene (Phe), anthracene (An), fluoranthene (Fluo), pyrene (Pyr), 11H-benzo[a]fluoranthene (11H-B[a]F), 11H-benzo[b]fluoranthene (11H-B[b]F), benzo[a]anthracene (B[a]A), chrysene (Chry), benzo[b]fluoranthene (B[b]F), benzo[k]fluoranthene (B[k]F), benzo[e]pyrene (B[e]P), benzo[a]pyrene (B[a]P), indeno[1,2,3-cd]pyrene (Ind), dibenz[a,h]anthracene (D[a,h]A), and benzo[g,h,i]perylene (B[g,h,i]P)

Table S7. PPMCC of PAH congeners in lake sediments collected at NHL. Any values that higher than 0.5 will be highlighted as bold.

|  | Phe | An | Fluo | Pyr | 11H-B[a]F | 11H-B[b]F | B[a]A | Chry | B[b]F | B[k]F | B[e]P | B[a]P | Ind | D[a,h]A | B[g,h,i]P |
| --- | --- | --- | --- | --- | --- | --- | --- | --- | --- | --- | --- | --- | --- | --- | --- |
| Phe | 1 |  |  |  |  |  |  |  |  |  |  |  |  |  |  |
| An | **.596**** | 1 |  |  |  |  |  |  |  |  |  |  |  |  |  |
| Fluo | **.546**** | **.929**** | 1 |  |  |  |  |  |  |  |  |  |  |  |  |
| Pyr | .402** | **.771**** | **.783**** | 1 |  |  |  |  |  |  |  |  |  |  |  |
| 11H-B[a]F | -.093 | .327** | .360** | .465** | 1 |  |  |  |  |  |  |  |  |  |  |
| 11H-B[b]F | -.065 | .**615**** | **.624**** | **.844**** | **.578**** | 1 |  |  |  |  |  |  |  |  |  |
| B[a]A | -.062 | .491** | .443** | **.790**** | .499** | **.929**** | 1 |  |  |  |  |  |  |  |  |
| Chry | -.129 | .308* | .276* | **.705**** | .451** | **.848**** | **.965**** | 1 |  |  |  |  |  |  |  |
| B[b]F | -.186 | .217 | .168 | **.623**** | .414** | **.786**** | **.932**** | **.985**** | 1 |  |  |  |  |  |  |
| B[k]F | -.149 | .230 | .173 | **.627**** | .392** | **.769**** | **.928**** | **.982**** | **.993**** | 1 |  |  |  |  |  |
| B[e]P | -.148 | .256* | .195 | **.643**** | .407** | **.794**** | **.940**** | **.985**** | **.995**** | **.993**** | 1 |  |  |  |  |
| B[a]P | .425** | **.506**** | **.626**** | **.639**** | .264* | .419** | .347** | .301* | .234 | .246 | .253* | 1 |  |  |  |
| Ind | .018 | .450** | .377** | **.755**** | .410** | **.842**** | **.954**** | **.952**** | **.944**** | **.951**** | **.960**** | .352** | 1 |  |  |
| D[a,h]A | -.046 | .170 | .169 | **.505**** | .375** | **.583**** | **.718**** | **.751**** | **.752**** | **.751**** | **.745**** | .219 | **.720**** | 1 |  |
| B[g,h,i]P | **.556**** | **.676**** | **.594**** | **.822**** | .238 | **.603**** | **.698**** | **.650**** | **.611**** | **.649**** | **.648**** | **.559**** | **.794**** | **.533**** | 1 |

| **. Correlation is significant at the 0.01 level (2-tailed). |
| --- |
| *. Correlation is significant at the 0.05 level (2-tailed). |
|  |

Where; phenanthrene (Phe), anthracene (An), fluoranthene (Fluo), pyrene (Pyr), 11H-benzo[a]fluoranthene (11H-B[a]F), 11H-benzo[b]fluoranthene (11H-B[b]F), benzo[a]anthracene (B[a]A), chrysene (Chry), benzo[b]fluoranthene (B[b]F), benzo[k]fluoranthene (B[k]F), benzo[e]pyrene (B[e]P), benzo[a]pyrene (B[a]P), indeno[1,2,3-cd]pyrene (Ind), dibenz[a,h]anthracene (D[a,h]A), and benzo[g,h,i]perylene (B[g,h,i]P)

Table S8. Varimax rotated component matrix of 15 PAH congeners from lake sediments (*n* = 73) collected at SKL. Any values that higher than 0.5 will be highlighted as bold.

|  | **Principal Component (PC)** | | | | |
| --- | --- | --- | --- | --- | --- |
|  | **PC1** | **PC2** | **PC3** | **PC4** | **PC5** |
| Phe | .160 | .276 | **.914** | .074 | -.097 |
| An | .183 | **.533** | **.813** | .067 | .060 |
| Fluo | **.509** | **.516** | **.573** | .304 | .047 |
| Pyr | **.843** | .322 | .396 | .099 | -.008 |
| 11H-B[a]F | .016 | **.927** | .261 | .074 | .081 |
| 11H-B[b]F | .042 | **.908** | .349 | .091 | .018 |
| B[a]A | .093 | **.864** | .259 | .209 | .090 |
| Chry | -.051 | .376 | **.838** | -.134 | .293 |
| B[b]F | **.829** | .107 | .089 | **.513** | -.020 |
| B[k]F | **.890** | .328 | .039 | .248 | .055 |
| B[e]P | .338 | .175 | -.064 | **.912** | .016 |
| B[a]P | .426 | **.723** | .364 | .146 | .026 |
| Ind | **.971** | .005 | .049 | .187 | .043 |
| D[a,h]A | .176 | .312 | .253 | **.649** | **.604** |
| B[g,h,i]P | **.986** | -.110 | .039 | -.033 | .034 |
| % of Variance | 55.0 | 23.6 | 9.33 | 5.48 | 2.06 |

Where; phenanthrene (Phe), anthracene (An), fluoranthene (Fluo), pyrene (Pyr), 11H-benzo[a]fluoranthene (11H-B[a]F), 11H-benzo[b]fluoranthene (11H-B[b]F), benzo[a]anthracene (B[a]A), chrysene (Chry), benzo[b]fluoranthene (B[b]F), benzo[k]fluoranthene (B[k]F), benzo[e]pyrene (B[e]P), benzo[a]pyrene (B[a]P), indeno[1,2,3-cd]pyrene (Ind), dibenz[a,h]anthracene (D[a,h]A), and benzo[g,h,i]perylene (B[g,h,i]P)

Table S9. Varimax rotated component matrix of 15 PAH congeners from lake sediments (*n* = 62) collected at NHL. Any values that higher than 0.5 will be highlighted as bold.

|  | **Principal Component (PC)** | | | | |
| --- | --- | --- | --- | --- | --- |
|  | **PC1** | **PC2** | **PC3** | **PC4** | **PC5** |
| Phe | -.192 | .489 | **.796** | -.128 | .175 |
| An | .157 | **.935** | .227 | .091 | .080 |
| Fluo | .087 | **.910** | .139 | .161 | .260 |
| Pyr | **.565** | **.681** | .173 | .158 | .300 |
| 11H-B[a]F | .281 | .241 | -.100 | **.894** | .079 |
| 11H-B[b]F | **.723** | **.564** | -.233 | .255 | .124 |
| B[a]A | **.902** | .367 | -.076 | .166 | .071 |
| Chry | **.962** | .170 | -.058 | .134 | .097 |
| B[b]F | **.981** | .067 | -.056 | .109 | .062 |
| B[k]F | **.983** | .071 | -.012 | .083 | .068 |
| B[e]P | **.981** | .101 | -.031 | .091 | .065 |
| B[a]P | .156 | .378 | .161 | .085 | **.891** |
| Ind | **.934** | .291 | .066 | .065 | .087 |
| D[a,h]A | **.780** | -.080 | .292 | .328 | .052 |
| B[g,h,i]P | **.618** | .486 | **.513** | -.057 | .229 |
| % of Variance | 61.3 | 20.2 | 6.96 | 3.94 | 3.39 |

Where; phenanthrene (Phe), anthracene (An), fluoranthene (Fluo), pyrene (Pyr), 11H-benzo[a]fluoranthene (11H-B[a]F), 11H-benzo[b]fluoranthene (11H-B[b]F), benzo[a]anthracene (B[a]A), chrysene (Chry), benzo[b]fluoranthene (B[b]F), benzo[k]fluoranthene (B[k]F), benzo[e]pyrene (B[e]P), benzo[a]pyrene (B[a]P), indeno[1,2,3-cd]pyrene (Ind), dibenz[a,h]anthracene (D[a,h]A), and benzo[g,h,i]perylene (B[g,h,i]P)


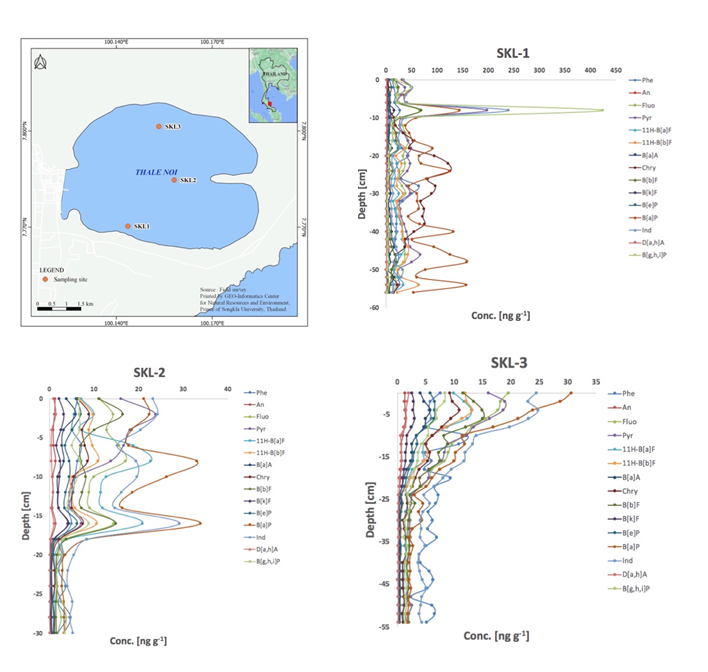


Fig. S1. Vertical profile of 15 PAH congeners in lake sediments collected at SKL.


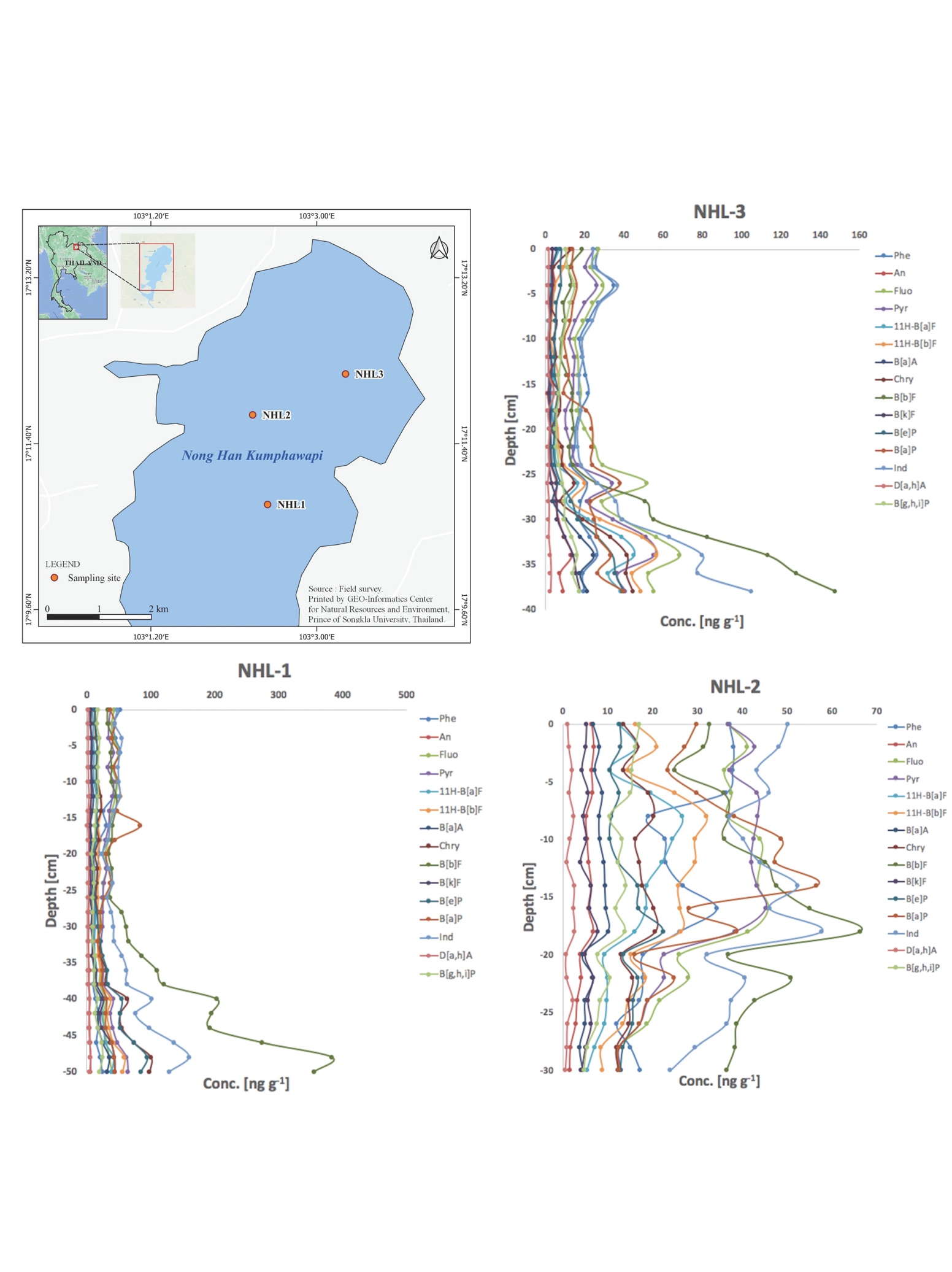


Fig. S2. Vertical profile of 15 PAH congeners in lake sediments collected at NHL.
